# Association of human *XPA* rs1800975 polymorphism and cancer susceptibility: an integrative analysis of 71 case–control studies

**DOI:** 10.1186/s12935-020-01244-5

**Published:** 2020-05-13

**Authors:** Maoxi Yuan, Chunmei Yu, Kuiying Yu

**Affiliations:** 1 Department of Thoracic Surgery, Linyi Central Hospital, No. 17 Jiankang Road, Yishui County, Linyi, Shandong 276400 People’s Republic of China; 2 First Department of Neurology, The First Hospital of Zibo, Zibo, Shandong 255200 People’s Republic of China

**Keywords:** XPA, Cancer, Polymorphism, Susceptibility

## Abstract

**Background:**

The objective of the present study is to comprehensively evaluate the impact of the rs1800975 A/G polymorphism within the human xeroderma pigmentosum group A (*XPA*) gene on susceptibility to overall cancer by performing an integrative analysis of the current evidence.

**Methods:**

We retrieved possible relevant publications from a total of six electronic databases (updated to April 2020) and selected eligible case–control studies for pooled assessment. *P*-values of association and odds ratio (OR) were calculated for the assessment of association effect. We also performed Begg’s test and Egger’s test, sensitivity analysis, false-positive report probability (FPRP) analysis, trial sequential analysis (TSA), and expression/splicing quantitative trait loci (eQTL/sQTL) analyses.

**Results:**

In total, 71 case–control studies with 19,257 cases and 30,208 controls from 52 publications were included for pooling analysis. We observed an enhanced overall cancer susceptibility in cancer cases compared with negative controls in the Caucasian subgroup analysis for the genetic models of allelic G vs. A, carrier G vs. A, homozygotic GG vs AA, heterozygotic AG vs. AA, dominant AG + GG vs. AA and recessive GG vs. AA + AG (*P *< 0.05, OR > 1). A similar positive conclusion was also detected in the “skin cancer” or “skin basal cell carcinoma (BCC)” subgroup analysis of the Caucasian population. Our FPRP analysis and TSA results further confirmed the robustness of the conclusion. However, our eQTL/sQTL data did not support the strong links of rs1800975 with the gene expression or splicing changes of *XPA* in the skin tissue. In addition, even though we observed a decreased risk of lung cancer under the homozygotic, heterozygotic and dominant models (*P *< 0.05, OR < 1) and an enhanced risk of colorectal cancer under the allelic, homozygotic, heterozygotic, dominant (*P *< 0.05, OR > 1), our data from FPRP analysis and another pooling analysis with only the population-based controls in the Caucasian population did not support the strong links between the *XPA* rs1800975 A/G polymorphism and the risk of lung or colorectal cancer.

**Conclusions:**

Our findings provide evidence of the close relationship between the *XPA* rs1800975 A/G polymorphism and susceptibility to skin cancer in the Caucasian population. The potential effect of *XPA* rs1800975 on the risk of developing lung or colorectal cancer still merits the enrollment of larger well-scaled studies.

## Background

The nucleotide excision repair (NER) system participates in the removal of the bulky adducts of DNA lesions from the genome under environmental stimuli, such as UV irradiation, tobacco, alkylating agents or pollutants, and xeroderma pigmentosum group A (XPA) acts as an essential NER member [1, 2]. XPA protein, as a zinc finger DNA binding protein and an important damage verifier, can bind the NER core repair factors to identify the damage site of the DNA substrate [2–4]. Abnormal DNA repair mechanisms or mutated NER proteins are involved in the process of mutagenesis and oncogenesis and are often linked to a group of clinical disorders [1, 2]. The human *XPA* rs1800975 T/C polymorphism is a common single nucleotide polymorphism (SNP) in the 5′-untranslated region of the *XPA* gene [5]. In the present study, we are interested in comprehensively exploring the possible effect of the *XPA* rs1800975 genetic variant on the susceptibility to different cancer diseases, such as skin cancer, lung cancer, breast cancer, esophageal cancer, gastric cancer, colorectal cancer or endometrial cancer.

There are different reports with distinct conclusions regarding the genetic relationship between the *XPA* rs1800975 polymorphism and cancer susceptibility in varied populations. For example, the *XPA* rs1800975 polymorphism was reported to be related to the risk of lung cancer in Norwegian [6], Germany [7, 8] or Korean populations [9] but not in patients from Belgium [10] or the USA [11]. These results merit a comprehensive evaluation by means of a meta-analysis.

To the best of our knowledge, to date, only two meta-analyses regarding the association between the *XPA* rs1800975 polymorphism and susceptibility to overall cancer diseases have been previously reported in 2012 [12, 13]. Nevertheless, no more than 36 case–control studies were enrolled for the prior meta-analysis. Therefore, we performed an updated comprehensive meta-analysis in 2020 based on the guidelines of preferred reporting items for systematic reviews and meta-analyses (PRISMA) [14]. In total, 71 case–control studies following the principle of Hardy–Weinberg equilibrium (HWE) were enrolled for pooling, and a series of stratified analyses, Begg’s test, Egger’s test, sensitivity analysis, FPRP analysis and TSA test, expression pattern, eQTL and sQTL analysis were conducted.

## Methods

### Database retrieval

Potentially relevant publications from six online databases, including PubMed, Excerpta Medica Database (EMBASE), Cochrane, China National Knowledge Infrastructure (CNKI), WANFANG and VIP, were retrieved until April 8, 2020. We did not set up any geographical or language restrictions for publications. Additional file 1: Table S1 shows our specific search terms during the database retrieval.

### Screening criteria

The articles were then screened and evaluated for eligibility, according to our screening criteria. The inclusion criteria were as follows: genotypic frequency data for the *XPA* rs1800975 polymorphism in both cases and controls. The exclusion criteria included duplicate information; cell, plant or animal assay data; other diseases, genes or SNPs; review, meeting or meta-analysis; lack of normal control; lack of full genotypic data; and the genotypic distribution in controls was not in line with HWE.

### Data extraction and quality evaluation

We utilized a table to independently extract the basic information, including first author, publication year, country, race, genotypic distribution, cancer type, control source, genotyping method, genotype frequency, and sample size. Possible disagreements were resolved by full discussion, and missing data were obtained by attempting to contact the corresponding author via e-mail. The P value of HWE in controls was obtained by the Chi square test. We evaluated the methodological quality of studies using the criteria of the Newcastle–Ottawa quality assessment scale (NOS) with a score ranging from one to nine. If the NOS score was less than five, the study was considered to be of poor quality.

### Heterogeneity and association test

If the I^2^ value (variation in ORs attributable to heterogeneity) > 50% and the *P*-value of heterogeneity < 0.05, we adopted a random-effect model for the test of association. Otherwise, a fixed-effect model was used, owing to the absence of significant interstudy heterogeneity. *P*-values of association, OR and 95% CI (confidence interval) were calculated for the allelic (G vs. A), carrier (G vs. A), homozygotic (GG vs AA), heterozygotic (AG vs. AA), dominant (AG + GG vs. AA) and recessive (GG vs. AA + AG) models. In addition, subgroup analyses for race, control source and genotyping method were conducted. In the subgroup analysis, a minimum of three case–control studies should be included to obtain a relatively scientific and reliable conclusion.

### Publication bias assessment

Begg’s test and Egger’s test were carried out for the quantitative evaluation of potential publication bias. We finally obtained the *P*-values for Begg’s test and Egger’s test, Begg’s funnel plot (pseudo 95% confidence limit) and Egger’s publication bias plot. If there is a basic symmetrical funnel plot and yielded *P*-values were larger than 0.05, the absence of significant publication bias was suggested.

### Data sensitivity

We also conducted sensitivity analyses under the above six genetic models. After the sequential removal of each case–control study, the obvious change in the estimates showed the lack of statistical stability. STATA 12.0 software (StataCorp, College Station, USA) was used for the above statistical analysis.

### False-positive report probability test

As the relevant information of former studies [15–17], a false-positive report probability (FPRP) test was carried out for the assessment of the true genetic relationship probability under the parameters of FPRP threshold value with 0.2, power OR with 1.5, and prior probability levels with “0.25, 0.1, 0.01, 0.001, 0.0001, 0.00001″. If the FPRP value < 0.2 under the prior probability level of 0.1, a worthy outcome between *XPA* rs1800975 and cancer risk was considered.

### Trial sequential analysis

We applied a trial sequential analysis (TSA) approach to adjust random and systematic error risk and provided the optimal sample size for pooling by means of TSA viewer software (Copenhagen Trial Unit, Copenhagen), similar to several reported studies [17–19]. The TSA plot with a two-sided boundary type was obtained by the parameters of type I error probability with 5%, statistical test power with 80%, and relative risk reduction with 20%. For the genetic model of AG + GG vs. AA, if the cumulative Z-curve crossed the TSA monitoring boundary and touched the line of required information size, the power of the results with robustness was regarded.

### Expression pattern analysis

Based on the dataset of GTEx (Genotype-Tissue Expression) analysis release V8 (dbGaP accession phs000424.v8.p2) [20], we analyzed the expression profile of *XPA* gene (ENSG0000136936.10) across multiple tissues, such as heart, brain, lung, stomach or colon. Log_10_ [TPM (Transcripts Per Million) +1] was utilized for scale. Besides, we applied the TIMER (Tumor Immune Estimation Resource) approach [21] to compare the expression difference of the *XPA* gene between tumor and adjacent normal tissues across all TCGA (The Cancer Genome Atlas) tumors. Wilcoxon test was used for the assessment of statistical significance. The results were visualized by the violin plot or box-plot.

### The eQTL and sQTL analysis

Based on the dataset of GTEx [20], we also analyzed the “Significant Single-Tissue” eQTL (expression quantitative trait loci) and sQTL (splicing quantitative trait loci) in all tissues, for the *XPA* gene and the rs1800975 SNP. The values of sample number, NES (Normalized Effect Size), p-value, m-value were obtained. When m-value was larger than 0.9, an eQTL effect was considered [22]. The violin plots of eQTL and sQTL, and multi-tissue eQTL plots of the cross-tissue meta-analysis were provided, respectively. The normalized intron-excision ratio was used for the scale of sQTL.

## Results

### Enrolled case–control studies

**Fig. 1:**
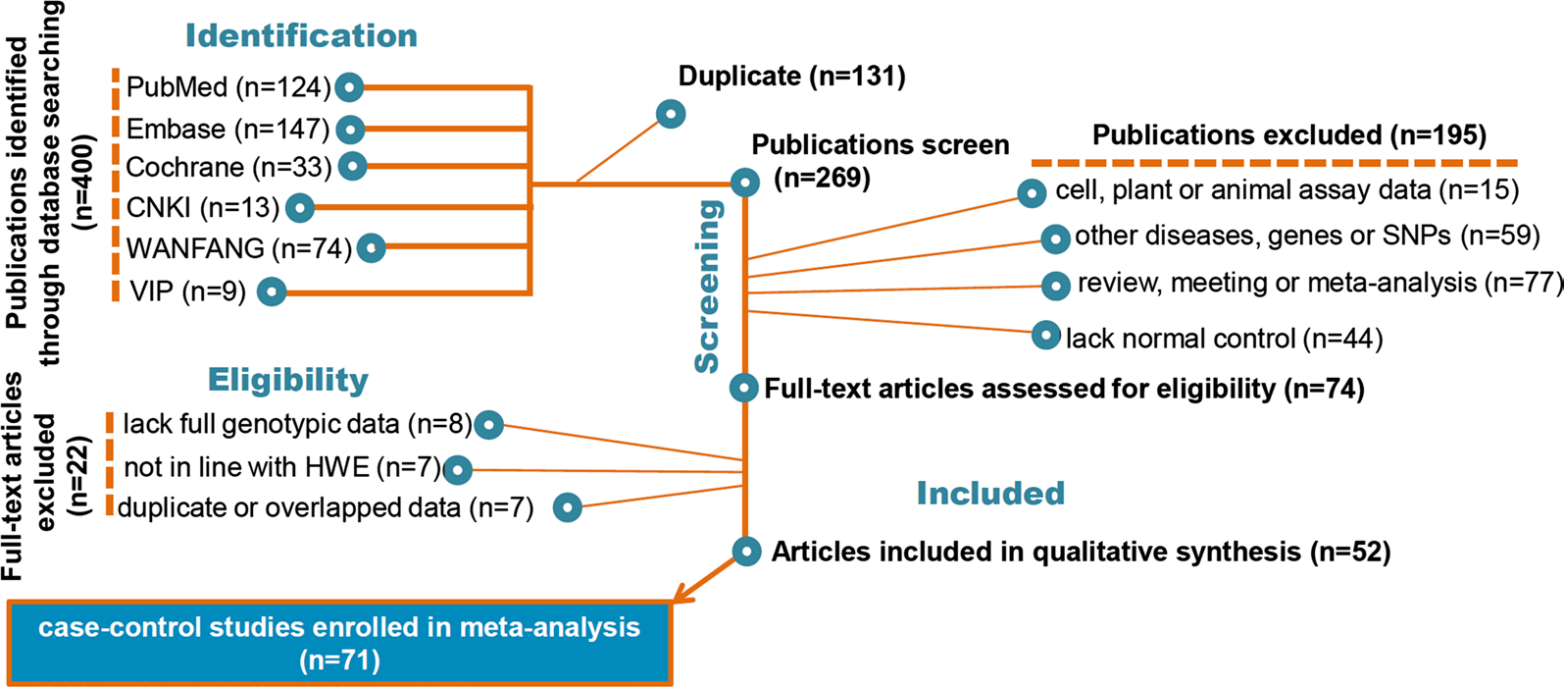
Schematic illustration of case–control identification in our meta-analysis

A schematic illustration of eligible case–control study selection is shown in Fig. 1. We initially obtained 400 publications from six databases. Then, duplicate publications were excluded, and the remaining 269 publications were screened. Of them, we further removed 195 publications using our screening criteria. A total of 22 full-text articles were also excluded due to “lack full genotypic data”, “not in line with HWE” or “duplicate or overlapped data”. We finally extracted a total of 71 case–control studies from 52 publications [6–11, 23–68] for our integrated analysis. Table 1 lists the main characteristics of the enrolled case–control studies with good methodological quality (NOS score ≥ 5).

**Table 1:** Characteristics of included case–control studies

First author, year [refs.]	NOS	Country/race	AA/AG/GG (case)	Cancer type	AA/AG/GG (control)	Control source	Genotyping method
Abbasi, 2009 [23]	8	Germany/Caucasian	30/109/107	Laryngeal cancer	72/281/291	PB	Real-time PCR
Akhmadishina, 2014 [24]	8	Russia/Caucasian	39/53/43	BC (Russian)	28/68/48	PB	PCR-RFLP
		Russia/Caucasian	18/35/23	BC (Tatar)	35/67/32	PB	PCR-RFLP
		Russia/Caucasian	7/16/3	BC (Bashkir)	22/35/13	PB	PCR-RFLP
		Russia/Caucasian	9/23/21	RCC (Russian)	28/68/48	PB	PCR-RFLP
		Russia/Caucasian	7/20/13	RCC (Tatar)	35/67/32	PB	PCR–RFLP
		Russia/Caucasian	3/5/4	RCC (Bashkir)	22/35/13	PB	PCR–RFLP
Applebaum, 2007 [25]	8	USA/Caucasian	95/345/428	skin BCC	101/325/347	PB	Taqman
		USA/Caucasian	72/268/322	skin SCC	101/325/347	PB	Taqman
Bau, 2007 [26]	7	China/Asian	38/84/32	oral cancer	29/53/23	HB	PCR-RFLP
Butkiewicz, 2004 [8]	7	Germany/Caucasian	23/94/93	LSCC	46/213/198	HB	Melting curves/PCR-RFLP
Chen, 2016 [27]	8	China/Asian	41/39/28	endometrial Cancer	35/45/30	PB	PCR-RFLP
Crew, 2007 [28]	7	USA/Caucasian	105/466/488	Breast cancer	137/477/488	PB	Taqman
De, 2007 [10]	8	Belgium/Caucasian	10/54/46	Lung cancer	10/54/45	PB	PCR-RFLP
Ding, 2016 [29]	8	China/Asian	44/66/20	Breast cancer	56/88/44	PB	PCR-RFLP
Ding, 2014 [30]	7	China/Asian	201/268/137	Breast cancer	157/299/177	HB	PCR-LDR
Doherty, 2011 [31]	6	USA/Mixed	67/297/339	Endometrial Cancer	66/320/328	PB	SNPlex/SNaPshot
Dong, 2008 [32]	9	China/Asian	86/120/47	GCA	162/322/128	PB	PCR-RFLP
Feng, 2008 [33]	7	China/Asian	85/83/28	Esophageal cancer	54/91/56	HB	PCR-RFLP
Liang, 2004 [34]	9	China/Asian	95/188/100	LSCC	204/462/221	PB	PCR-RFLP
		China/Asian	64/127/74	LA	204/462/221	PB	PCR–RFLP
		China/Asian	25/50/24	NSCLC	204/462/221	PB	PCR-RFLP
Ghanshela, 2020 [35]	7	India/Asian	24/60/16	bladder cancer	44/47/9	HB	PCR-RFLP
Gil, 2012 [36]	7	Poland/Caucasian	16/67/50	colorectal cancer	16/58/26	HB	PCR-RFLP
Guo, 2008 [37]	9	China/Asian	123/139/65	ESCC	162/322/128	PB	PCR-RFLP
Hall, 2007 [38]	6	Mixed/Caucasian	21/71/75	OSCC	98/375/297	HB	5′ exonuclease assay
		Mixed/Caucasian	15/42/54	Pharynx SCC	98/375/297	HB	5′ exonuclease assay
		Mixed/Caucasian	39/134/146	Laryngeal SCC	98/375/297	HB	5′ exonuclease assay
		Mixed/Caucasian	15/81/75	ESCC	125/451/398	HB	5′ exonuclease assay
Han, 2012 [39]	9	Korea/Asian	74/190/82	Breast cancer	103/169/89	PB	Illumina GoldenGate
Hansen, 2007 [40]	7	Denmark/Caucasian	31/187/176	Colorectal cancer	90/359/339	PB	Sequence dectection
Hsieh, 2010 [41]	6	China/Asian	33/87/38	Leiomyoma	35/84/37	HB	PCR-RFLP
Huang, 2007 [42]	9	China/Asian	59/69/22	Esophageal cancer	210/160/32	PB	PCR-RFLP
		China/Asian	65/60/20	Cardia gastric cancer	112/55/13	PB	PCR–RFLP
		China/Asian	77/57/12	Non-cardia gastric cancer	112/55/13	PB	PCR-RFLP
Jelonek, 2010 [43]	7	Poland/Caucasian	4/33/29	Colon cancer	17/70/46	PB	PCR-RFLP
		Poland/Caucasian	11/45/35	Breast cancer	48/168/142	PB	PCR-RFLP
Joshi, 2009 [44]	8	China/Asian	66^a^/61	Colon cancer	91^a^/52	PB	Taqman
		China/Asian	100^a^/75	Rectal cancer	109^a^/94	PB	Taqman
Lawania, 2019 [45]	6	India/Asian	82/59/4	Lung cancer	109/49/4	PB	PCR-RFLP
Liu, 2007 [46]	8	China/Asian	50/35/11	Esophageal cancer	38/47/11	PB	PCR-RFLP
Miller, 2006 [47]	8	USA/Caucasian	97/352/437	Skin BCC	101/340/355	PB	PCR-RFLP
		USA/Caucasian	74/277/331	Skin SCC	101/340/355	PB	PCR-RFLP
Palli, 2010 [48]	7	Italy/Caucasian	35/115/134	Gastric cancer	59/215/249	PB	Taqman
Pan, 2009 [49]	8	USA/Caucasian	35/166/179	Esophageal cancer	88/219/151	PB	PCR-RFLP
Park, 2002 [9]	9	Korea/Asian	60/160/45	Lung cancer	38/101/46	PB	PCR-RFLP
Paszkowska, 2013 [50]	6	Poland/Caucasian	78/294/306	Melanoma	93/255/240	PB	Taqman
Pesz, 2014 [51]	6	Poland/Caucasian	7/53/38	Skin BCC	16/58/26	PB	PCR-RFLP
Popanda, 2004 [7]	5	Germany/Caucasian	29/85/90	LA	46/213/198	HB	Rapid capillary PCR
Qian, 2011 [52]	9	China/Asian	163/272/146	NSCLC	131/301/171	PB	Taqman
		China/Asian	86/131/68	LSCC	131/301/171	PB	Taqman
		China/Asian	53/114/62	LA	131/301/171	PB	Taqman
Raaschou, 2008 [53]	7	Denmark/Caucasian	53/190/184	Lung cancer	90/355/335	PB	Taqman
Rafiq, 2016 [54]	7	India/Asian	181/170/99	ESCC	223/189/38	HB	PCR-RFLP
Sakoda, 2012 [11]	8	USA/Caucasian	71/326/320	Lung cancer	166/621/622	PB	GoldenGate/TaqMan
Tang, 2011 [55]	7	China/Asian	17/62/25	ALL	52/74/43	PB	MALDI-TOF-MS
Tao, 2018 [56]	6	China/Asian	111/197/85	Neuroblastoma	191/432/189	HB	Taqman
Vogel, 2005 [57]	7	Denmark/Caucasian	32107/117	Lung cancer	23/98/148	PB	Taqman
Weiss, 2005 [58]	8	USA/Mixed	29/147/195	Endometrial cancer	44/191/185	PB	SNaPshot
Wu, 2003 [59]	9	USA/others	20/13/17	Lung cancer	9/19/19	PB	PCR-RFLP
		USA/African	15/30/36	lung cancer	7/26/34	PB	PCR–RFLP
Xie, 2007 [60]	7	China/Asian	15/50/37	HCC	67/144/82	PB	PCR-RFLP
Zeng, 2013 [61]	8	China/Asian	29/73/37	Lung cancer	29/73/31	PB	PCR-RFLP
Zhang, 2006 [62]	7	China/Asian	91/82/33	Esophageal cancer	66/96/44	HB	PCR-RFLP
Zhao, 2018 [63]	8	China/Asian	22/45/22	Ovarian cancer	108/165/83	PB	Taqman
Zhen, 2012 [64]	9	China/Asian	107/145/99	Esophageal cancer	159/188/53	PB	PCR--RFLP
Zhu, 2015 [65]	8	China/Asian	78/111/109	Breast cancer	85/136/77	PB	Sequenom MassArray
Zhu, 2018 [66]	7	China/Asian	30/72/42	Wilms tumor	124/281/126	PB	Taqman
Zhu, 2005 [67]	7	China/Asian	84/133/93	Lung cancer	72/180/89	HB	PCR-RFLP
Zhu, 2008 [68]	8	China/Asian	69/69/50	ESCC	63/88/52	PB	PCR-RFLP
Zienolddiny, 2006 [6]	8	Norway/Caucasian	30/88/130	NSCLC	37/125/114	PB	Taqman

*Ref* Reference*, NOS* Newcastle–Ottawa quality assessment Scale, *BC* bladder cancer, *RCC* renal cell carcinoma, *SCC* squamous cell carcinoma, *BCC* basal cell carcinoma, *LSCC* lung squamous cell carcinoma, *GCA* gastric cardiac adenocarcinoma, *LA* lung adenocarcinoma, *NSCLC* non-small cell lung cancer, *ESCC* esophageal squamous cell carcinoma, *OSCC* oral squamous cell carcinoma, *ALL* acute lymphoblastic leukemia, *HCC* hepatocellular carcinoma, *PB* population-based control, *HB* hospital-based control, *PCR* polymerase chain reaction, *PCR*-*RFLP* PCR-restriction fragment length polymorphism, *PCR*-*LDR* PCR-ligase detection reaction, *MALDI*-*TOF*-*MS* matrix-assisted laser desorption/Ionization time of flight mass spectrometry

^a^ The combined frequency of AA + AG genotypes

### Overall meta-analysis results

As shown in Table 2, our overall meta-analysis enrolled a total of 71 case–control studies with 19,257 cases and 30,208 controls under the recessive model (GG vs. AA + AG) and 69 case–control studies with 19,039 cases and 29,707 controls under the other genetic models. The heterogeneity under the carrier G vs. A model (Table 2, I^2^ = 22.3%, *P* = 0.056) led to the utilization of a fixed-effects pooling model, and a random-effects pooling model was applied for others. For the pooling results shown in Table 2, a statistically significant difference in the susceptibility to cancer between cases and controls was detected under the allelic (*P* = 0.026, OR = 1.07), carrier (*P* = 0.009, OR = 1.04) and recessive (*P* = 0.001, OR = 1.12) genetic models. However, negative results were observed under other models (Table 2, *P *> 0.05). We failed to obtain evidence regarding the relationship between the *XPA* rs1800975 polymorphism and the overall risk of cancer in the overall population.

**Table 2:** Overall meta-analysis and publication bias data

Models	Study number (case/control)	Heterogeneity		Association				Bias	
		I^2^	*P*^a^	Fixed/Random	OR (95% CI)	z	*P*^b^	*P*^c^	*P*^d^
Allelic model (G vs. A)	69 (19,039/29,707)	72.0%	< 0.001	Random	1.07 (1.01–1.13)	2.23	0.026	0.645	0.719
Carrier model (G vs. A)	69 (19,039/29,707)	22.3%	0.056	Fixed	1.04 (1.01–1.08)	2.62	0.009	0.637	0.727
Homozygotic model (GG vs AA)	69 (19,039/29,707)	68.9%	< 0.001	Random	1.12 (1.00–1.25)	1.92	0.054	0.404	0.476
Heterozygotic model (AG vs. AA)	69 (19,039/29,707)	54.2%	< 0.001	Random	1.00 (0.92–1.09)	< 0.01	0.996	0.303	0.215
Dominant model (AG + GG vs. AA)	69 (19,039/29,707)	66.0%	< 0.001	Random	1.05 (0.96–1.15)	1.02	0.307	0.393	0.231
Recessive model (GG vs. AA + AG)	71 (19,257/30,208)	57.5%	< 0.001	Random	1.12 (1.04–1.20)	3.19	0.001	0.481	0.753

*OR* odds ratio, *CI* confidence interval, ^a^*P*-value of Cochrane’s Q statistic for the assessment of heterogeneity, ^b^*P*-value of association, ^c^*P*-value of Begg’s test, ^d^*P*-value of Egger’s test

### Subgroup analysis results

Next, we conducted a series of subgroup meta-analyses stratified by race, control source and genotyping method. As shown in Table 3, an increased cancer risk in cases was observed compared with negative controls in the Caucasian subgroup analysis under the models of allelic G vs. A (*P* < 0.001, OR = 1.12), carrier G vs. A (*P* = 0.001, OR = 1.08), homozygotic GG vs AA (*P* < 0.001, OR = 1.24), heterozygotic AG vs. AA (*P* = 0.046, OR = 1.10), dominant AG + GG vs. AA (*P* = 0.004, OR = 1.16) and recessive GG vs. AA + AG (*P* < 0.001, OR = 1.16). A similar positive conclusion was detected in the subgroup analysis of the “population-based control, PB” under the allelic, carrier, homozygotic and recessive models (Table 3, *P* < 0.05, OR > 1). For the PCR-RFLP subgroup analysis, we only observed an increased risk of cancer in the carrier (Table 3, *P* = 0.016, OR = 1.06) and recessive (*P* = 0.018, OR = 1.16) models.

**Table 3:** Subgroup analyses by race, control source and genotyping assay

Models	Factor-subgroup	Study number (case/control)	OR (95% CI)	z	*P*
Allelic model (G vs. A)	Race-Asian	34 (7941/12,945)	1.03 (0.93–1.13)	0.59	0.558
	Race-Caucasian	31 (9809/1,5669)	1.12 (1.06–1.18)	4.01	< 0.001
	Control source-PB	53 (15,067/22,560)	1.08 (1.02–1.14)	2.51	0.012
	Control source-HB	16 (3888/7302)	1.03 (0.89–1.19)	0.41	0.680
	Genotyping assay-PCR-RFLP	40 (7785/11,636)	1.08 (0.98–1.19)	1.59	0.111
Carrier model (G vs. A)	Race-Asian	34 (7941/12,945)	1.00 (0.95–1.05)	0.04	0.964
	Race-Caucasian	31 (9809/1,5669)	1.08 (1.03–1.13)	3.46	0.001
	Control source-PB	53 (15,067/22,560)	1.05 (1.02–1.09)	2.84	0.005
	Control source-HB	16 (3888/7302)	1.01 (0.94–1.08)	0.23	0.815
	Genotyping assay-PCR-RFLP	40 (7785/11,636)	1.06 (1.01–1.12)	2.41	0.016
Homozygotic model (GG vs. AA)	Race-Asian	34 (7941/12,945)	1.05 (0.87–1.26)	0.48	0.629
	Race-Caucasian	31 (9809/1,5669)	1.24 (1.10–1.39)	3.57	<0.001
	Control source-PB	53 (15,067/22,560)	1.15 (1.02–1.29)	2.30	0.022
	Control source-HB	16 (3888/7302)	1.04 (0.78–1.39)	0.25	0.805
	Genotyping assay-PCR-RFLP	40 (7785/11,636)	1.16 (0.96–1.41)	1.52	0.129
Heterozygotic model (AG vs. AA)	Race-Asian	34 (7941/12,945)	0.97 (0.85–1.09)	0.55	0.584
	Race-Caucasian	31 (9809/1,5669)	1.10 (1.00–1.20)	2.00	0.046
	Control source-PB	53 (15,067/22,560)	1.04 (0.85–1.14)	0.87	0.385
	Control source-HB	16 (3888/7302)	0.87 (0.74–1.03)	1.63	0.103
	Genotyping assay-PCR-RFLP	40 (7785/11,636)	1.00 (0.88–1.14)	0.02	0.589
Dominant model (AG + GG vs. AA)	Race-Asian	34 (7941/12,945)	1.00 (0.87–1.14)	0.02	0.988
	Race-Caucasian	31 (9809/1,5669)	1.16 (1.05–1.28)	2.86	0.004
	Control source-PB	53 (15,067/22,560)	1.09 (0.98–1.20)	1.64	0.101
	Control source-HB	16 (3888/7302)	0.94 (0.77–1.15)	0.62	0.535
	Genotyping assay-PCR-RFLP	40 (7785/11,636)	1.06 (0.92–1.22)	0.78	0.434
Recessive model (GG vs. AA + AG)	Race-Asian	36 (8243/13,291)	1.08 (0.94–1.22)	1.09	0.276
	Race-Caucasian	31 (9809/1,5669)	1.16 (1.08–1.24)	4.18	< 0.001
	Control source-PB	55 (15,369/22,906)	1.12 (1.04–1.19)	3.11	0.002
	Control source-HB	16 (3888/7302)	1.12 (0.92–1.37)	1.17	0.240
	Genotyping assay-PCR-RFLP	40 (7785/11,636)	1.16 (1.03–1.32)	2.37	0.018

*OR* odds ratio, *CI* confidence interval, *PB* population-based control, *HB* hospital-based control, *PCR*-*RFLP*. PCR-restriction fragment length polymorphism

As shown in Tables 4 and 5, compared with controls, a decreased lung cancer risk was detected in cases under the GG vs AA (*P *= 0.032, OR = 0.87), AG vs. AA (*P *= 0.014, OR = 0.86), AG + GG vs. AA (*P* = 0.021, OR = 0.87) models, but not allelic G vs. A (*P *= 0.155), carrier G vs. A (*P *= 0.345), and GG vs. AA + AG (*P *= 0.755) models. For the subgroup of digestive system cancer, a positive association was detected under the carrier (Table 4, *P *= 0.013, OR = 1.09) and recessive (Table 5, *P* = 0.025, OR = 1.26) models. Moreover, we observed an enhanced risk of colorectal cancer under allelic (Table 4, *P* = 0.021, OR = 1.20), homozygotic (*P* = 0.007, OR = 1.68), heterozygotic (Table 5, *P* = 0.041, OR = 1.46), and dominant (*P* = 0.016, OR = 1.54) conditions, implying the potential effect of the AG genotype of *XPA* rs1800975 on the risk of colorectal cancer.

**Table 4:** Subgroup analyses by cancer type under the allelic, carrier and homozygotic models

Models	Subgroup	Study number (case/control)	OR (95% CI)	z	*P*
Allelic model (G vs. A)	Lung cancer	19 (5004/9162)	0.95 (0.8–1.02)	1.42	0.155
	LSCC	3 (878/19,47)	0.91 (0.77–1.08)	1.08	0.282
	NSCLC	3 (928/1766)	1.00 (0.75–1.34)	< 0.01	0.999
	LA	3 (968/1947)	0.98 (0.86–1.11)	0.35	0.729
	Breast cancer	6 (2530/2940)	1.00 (0.83–1.20)	< 0.01	0.998
	Digestive system cancer	18 (4038/6811)	1.13 (0.96–1.34)	1.48	0.138
	Esophageal cancer	10 (2515/4002)	1.06 (0.82–1.39)	0.47	0.642
	ESCC	4 (1136/2239)	1.09(0.76–1.56)	0.45	0.654
	Gastric cancer	4 (828/1495)	1.14 (0.83–1.58)	0.82	0.412
	Colorectal cancer	3 (593/1021)	1.20 (1.03–1.40)	2.31	0.021
	Reproductive system cancer	5 (1429/1756)	1.10 (1.98–1.24)	1.54	0.123
	Endometrial cancer	3 (1182/1244)	1.09 (0.89–1.33)	0.86	0.390
	Head and neck cancer	4 (886/2289)	1.08 (0.96–1.22)	1.34	0.179
	Skin cancer	6 (3874/3826)	1.17 (1.09–1.25)	4.60	< 0.001
	Skin BCC	3 (1852/1669)	1.18 (1.07–1.31)	3.23	0.001
Carrier model (G vs. A)	Lung cancer	19 (5004/9162)	0.97 (0.92–1.03)	0.94	0.345
	LSCC	3 (878/19,47)	0.94 (0.83–1.08)	0.87	0.386
	NSCLC	3(928/1766)	0.97 (0.84–1.12)	0.42	0.675
	LA	3 (968/1947)	0.99 (0.86–1.14)	0.14	0.891
	Breast cancer	6 (2530/2940)	1.00 (0.91–1.10)	0.03	0.977
	Digestive system cancer	18 (4038/6811)	1.09 (1.02–1.17)	2.49	0.013
	Esophageal cancer	10 (2515/4002)	1.09 (1.00–1.19)	2.00	0.046
	ESCC	4 (1136/2239)	1.06 (0.93–1.20)	0.88	0.379
	Gastric cancer	4 (828/1495)	1.04 (0.89–1.20)	0.47	0.637
	Colorectal cancer	3 (593/1021)	1.12 (0.94–1.33)	1.29	0.199
	Reproductive system cancer	5 (1429/1756)	1.07 (0.95–1.21)	1.15	0.251
	Endometrial cancer	3 (1182/1244)	1.08 (0.94–1.24)	1.07	0.285
	Head and neck cancer	4 (886/2289)	1.07 (0.93–1.22)	0.93	0.355
	Skin cancer	6 (3874/3826)	1.12 (1.03–1.21)	2.82	0.005
	Skin BCC	3 (1852/1669)	1.13 (1.01–1.26)	2.05	0.040
Homozygotic model (GG vs. AA)	Lung cancer	19 (5004/9162)	0.87 (0.77–0.99)	2.15	0.032
	LSCC	3 (878/19,47)	0.81 (0.59–1.12)	1.27	0.206
	NSCLC	3 (928/1766)	0.91 (0.59–1.41)	0.42	0.677
	LA	3 (968/1947)	0.92 (0.71–1.18)	0.66	0.512
	Breast cancer	6 (2530/2940)	1.01 (0.70–1.45)	0.06	0.954
	Digestive system cancer	18 (4038/6811)	1.35 (0.96–1.89)	1.72	0.086
	Esophageal cancer	10 (2515/4002)	1.25 (0.74–2.11)	0.82	0.410
	ESCC	4 (1136/2239)	1.31 (0.61–2.81)	0.69	0.489
	Gastric cancer	4 (828/1495)	1.13 (0.67–1.93)	0.46	0.648
	Colorectal cancer	3 (593/1021)	1.68 (1.15–2.44)	2.70	0.007
	Reproductive system cancer	5 (1429/1756)	1.14 (0.90–1.44)	1.05	0.295
	Endometrial cancer	3 (1182/1244)	1.12 (0.78–1.60)	0.61	0.541
	Head and neck cancer	4 (886/2289)	1.09 (0.85–1.41)	0.67	0.503
	Skin cancer	6 (3874/3826)	1.36 (1.17–1.57)	4.11	< 0.001
	Skin BCC	3 (1852/1669)	1.40 (1.03–1.89)	2.17	0.030

*OR* odds ratio, *CI* confidence interval, *LSCC* lung squamous cell carcinoma, *NSCLC* non-small cell lung cancer, *LA* lung adenocarcinoma

*ESCC* esophageal squamous cell carcinoma, *BCC* basal cell carcinoma

**Table 5:** Subgroup analyses by cancer type under the heterozygotic, dominant and recessive models

Models	Subgroup	Study number (case/control)	OR (95% CI)	z	*P*
Heterozygotic model (AG vs. AA)	Lung cancer	19 (5004/9162)	0.86 (0.76–0.97)	2.46	0.014
	LSCC	3 (878/19,47)	0.79 (0.64–0.97)	2.23	0.026
	NSCLC	3 (928/1766)	0.78 (0.62–0.97)	2.19	0.029
	LA	3 (968/1947)	0.84 (0.67–1.06)	1.45	0.147
	Breast cancer	6 (2530/2940)	1.04 (0.79–1.38)	0.30	0.761
	Digestive system cancer	18 (4038/6811)	1.05 (0.85–1.30)	0.48	0.634
	Esophageal cancer	10 (2515/4002)	0.93 (0.70–1.23)	0.52	0.602
	ESCC	4 (1136/2239)	0.88 (0.58–1.34)	0.59	0.554
	Gastric cancer	4 (828/1495)	1.14 (0.71–1.82)	0.54	0.589
	Colorectal cancer	3 (593/1021)	1.46 (1.02–2.11)	2.04	0.041
	Reproductive system cancer	5 (1429/1756)	1.02 (0.81–1.28)	0.17	0.867
	Endometrial cancer	3 (1182/1244)	0.94 (0.72–1.23)	0.45	0.656
	Head and neck cancer	4 (886/2289)	0.95 (0.74–1.22)	0.39	0.694
	Skin cancer	6 (3874/3826)	1.18 (1.02–1.36)	2.18	0.029
	Skin BCC	3 (1852/1669)	1.14 (0.92–1.42)	1.17	0.241
Dominant model (AG + GG vs. AA)	Lung cancer	19 (5004/9162)	0.87 (0.77–0.98)	2.30	0.021
	LSCC	3 (878/19,47)	0.80 (0.63–1.01)	1.86	0.062
	NSCLC	3 (928/1766)	0.83 (0.64–1.07)	1.46	0.145
	LA	3 (968/1947)	0.88 (0.71–1.09)	1.20	0.230
	Breast cancer	6 (2530/2940)	1.04 (0.78–1.40)	0.28	0.782
	Digestive system cancer	18 (4038/6811)	1.14 (0.89–1.44)	1.04	0.297
	Esophageal cancer	10 (2515/4002)	1.01 (0.72–1.43)	0.06	0.953
	ESCC	4 (1136/2239)	0.99 (0.60–1.65)	0.02	0.982
	Gastric cancer	4 (828/1495)	1.16 (0.71–1.89)	0.58	0.561
	Colorectal cancer	3 (593/1021)	1.54 (1.08–2.20)	2.41	0.016
	Reproductive system cancer	5 (1429/1756)	1.07 (0.87–1.32)	0.63	0.528
	Endometrial cancer	3 (1182/1244)	1.01 (0.75–1.37)	0.09	0.926
	Head and neck cancer	4 (886/2289)	1.02 (0.81–1.29)	0.16	0.873
	Skin cancer	6 (3874/3826)	1.27 (1.10–1.45)	3.36	0.001
	Skin BCC	3 (1852/1669)	1.25 (1.00–1.56)	1.98	0.048
Recessive model (GG vs. AA + AG)	Lung cancer	19 (5004/9162)	0.99 (0.90–1.08)	0.31	0.755
	LSCC	3 (878/19,47)	0.97 (0.81–1.16)	0.35	0.726
	NSCLC	3 (928/1766)	1.08 (0.72–1.62)	0.39	0.700
	LA	3 (968/1947)	1.05 (0.87–1.27)	0.51	0.613
	Breast cancer	6 (2530/2940)	0.98 (0.77–1.24)	0.20	0.842
	Digestive system cancer	20 (4340/7157)	1.26 (1.03–1.54)	2.24	0.025
	Esophageal cancer	10 (2515/4002)	1.28 (0.89–1.83)	1.32	0.186
	ESCC	4 (1136/2239)	1.35 (0.81–2.27)	1.15	0.249
	Gastric cancer	4 (828/1495)	1.05 (0.79–1.40)	0.33	0.739
	Colorectal cancer	5 (895/1367)	1.22 (0.95–1.56)	1.59	0.111
	Reproductive system cancer	5 (1429/1756)	1.16 (1.00–1.34)	1.91	0.056
	Endometrial cancer	3 (1182/1244)	1.18 (0.97–1.44)	1.65	0.098
	Head and neck cancer	4 (886/2289)	1.15 (0.94–1.41)	1.39	0.165
	Skin cancer	6 (3874/3826)	1.20 (1.09–1.31)	3.93	< 0.001
	Skin BCC	3 (1852/1669)	1.23 (1.07–1.40)	2.99	0.003

*OR* odds ratio, *CI* confidence interval, *LSCC* lung squamous cell carcinoma, *NSCLC* non-small cell lung cancer, *LA* lung adenocarcinoma

*ESCC* esophageal squamous cell carcinoma, *BCC* basal cell carcinoma

Interestingly, as shown in Tables 4 and 5, we detected a significant difference between skin cancer cases and controls under the allelic (*P* < 0.001, OR = 1.17), carrier (*P* = 0.005, OR = 1.12), homozygotic (*P* < 0.001, OR = 1.36), heterozygotic (*P* = 0.029, OR = 1.18), dominant (*P* = 0.001, OR = 1.27), and recessive (*P* < 0.001, OR = 1.20) models. There was a similar positive association in the “skin BCC” subgroup under the allelic, carrier, homozygotic, dominant, and recessive models (all *P *< 0.05, OR > 1). These data suggested that *XPA* rs1800975 may be associated with a high susceptibility to skin cancer, especially skin BCC.

**Fig. 2:**
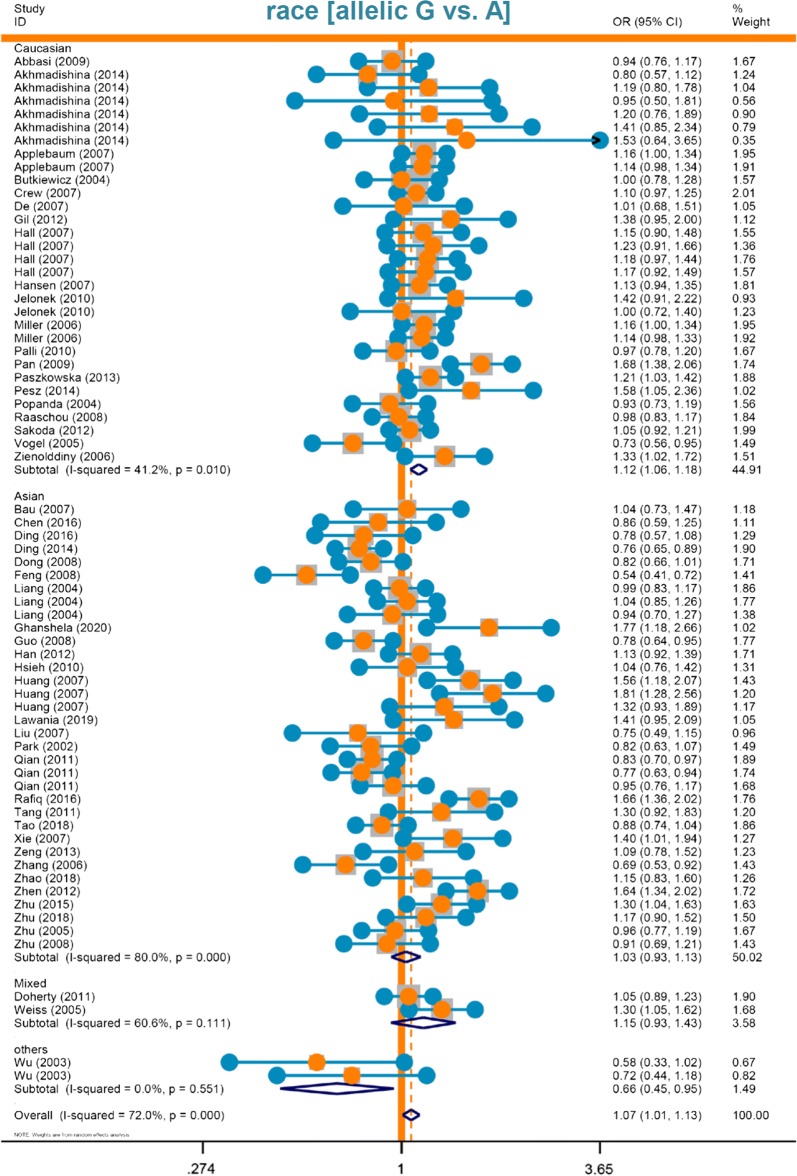
Forest plot data of subgroup analysis by race (allelic model)

**Fig. 3:**
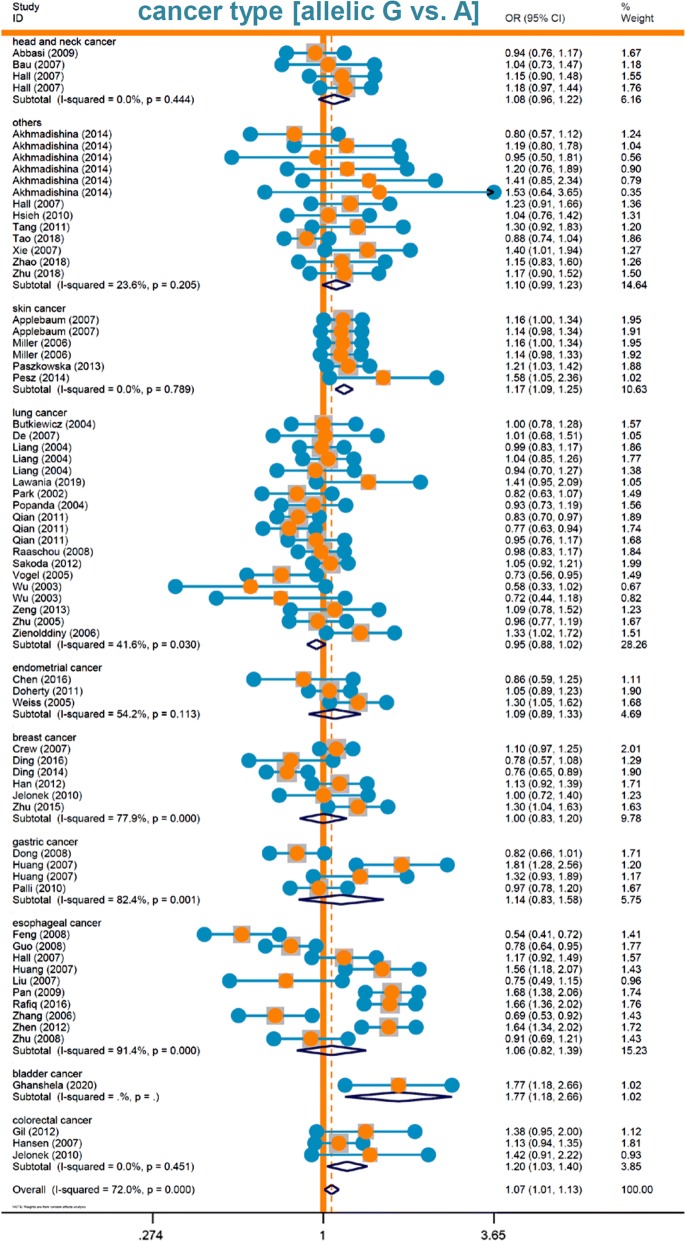
Forest plot data of subgroup analysis by cancer type (allelic model)

There were no significant differences between cases and controls in the majority of comparisons (Tables 2, 3, 4, *P* > 0.05), indicating that *XPA* rs1800975 does not seem to contribute to the risk of specific cancer types, such as breast cancer, esophageal cancer, gastric cancer, reproductive system cancer, endometrial cancer, or head and neck cancer. Forest plots of subgroup analyses by race (Fig. 2 of allelic model; Additional file 2: Fig. S1 of carrier model; Additional file 3: Fig. S2 of dominant model), control source (Additional file 4: Fig. S3 of allelic model; Additional file 5: Fig. S4 of carrier model; Additional file 6: Fig. S5 of dominant model), and cancer type (Fig. 3 of allelic model; Additional file 7: Fig. S6 of homozygotic model; Additional file 8: Fig. S7 of heterozygotic model; Additional file 9: Fig. S8 of dominant model) are presented as examples.

### FRAP and TSA results

To strengthen our results in the subgroup analysis of “lung cancer”, “colorectal cancer”, and “skin cancer”, we performed the FPRP test. As shown in Table 6, under the 0.1 prior probability level, the FPRP value for lung cancer was less than 0.20 under the heterozygotic and dominant models but not the homozygotic model, suggesting the lack of notable associations. We found that the subjects in different populations or the mixed source-based controls were included for the pooling analysis of lung cancer. Considering the above positive results in the subgroup of “Caucasian” and “PB”, we also performed another pooling analysis limited to the Caucasian population. As shown in Additional file 1: Table S2, when we only included the Caucasian subjects for the pooling analysis, we did not observe positive conclusions (all *P *> 0.05). A similar negative conclusion was further detected in the meta-analysis using PB-based controls in the Caucasian population (Additional file 1: Table S3, *P *> 0.05). Collectively, this evidence did not support the strong association between lung cancer risk and *XPA* rs1800975.

**Table 6:** FPRP values for the association between *XPA* rs1800975 and the risk of lung, skin, and colorectal cancers

Cancer type	Model	OR (95% CI)	P*	Prior probability level					
				0.25	0.1	0.01	0.001	0.0001	0.00001
Lung cancer	Homozygotic model (GG vs AA)	0.87 (0.77–0.99)	0.035	*0.094*	0.238	0.774	0.972	0.997	1.000
	Heterozygotic model (AG vs. AA)	0.86 (0.76–0.97)	0.014	*0.040*	*0.112*	0.582	0.933	0.993	0.999
	Dominant model (AG + GG vs. AA)	0.87 (0.77–0.98)	0.022	*0.062*	*0.164*	0.684	0.956	0.995	1.000
Colorectal cancer	Allelic model (G vs. A)	1.20 (1.03–1.40)	0.020	*0.058*	*0.156*	0.670	0.953	0.995	1.000
	Homozygotic model (GG vs AA)	1.68 (1.15–2.44)	0.006	*0.065*	*0.174*	0.698	0.959	0.996	1.000
	Heterozygotic model (AG vs. AA)	1.46 (1.02–2.11)	0.044	*0.191*	0.415	0.887	0.987	0.999	1.000
	Dominant model (AG + GG vs. AA)	1.54 (1.08–2.20)	0.018	*0.107*	0.264	0.798	0.976	0.997	1.000
Skin cancer	Allelic model (G vs. A)	1.17 (1.09–1.25)	< 0.001	**<***0.001*	**<***0.001*	**<***0.001*	**<***0.001*	**<***0.001*	0.247
	Carrier model (G vs. A)	1.12 (1.03–1.21)	0.004	*0.012*	*0.035*	0.286	0.802	0.976	0.998
	Homozygotic model (GG vs AA)	1.36 (1.17–1.57)	< 0.001	**<***0.001*	**<***0.001*	*0.003*	*0.029*	0.229	0.748
	Heterozygotic model (AG vs. AA)	1.18 (1.02–1.36)	0.022	*0.063*	*0.167*	0.688	0.957	0.996	1.000
	Dominant model (AG + GG vs. AA)	1.27 (1.10–1.45)	< 0.001	*0.001*	*0.004*	*0.039*	0.291	0.804	0.976
	Recessive model (GG vs. AA + AG)	1.20 (1.09–1.31)	< 0.001	<* 0.001*	<* 0.001*	*0.005*	*0.044*	0.316	0.822

*OR* odds ratio, *CI* 95% confidence interval, P* P value in Chi square test for genotype frequency distributions. FPRP value < 0.2 in italics

With regard to colorectal cancer, we only observed that the FPRP value was less than 0.20 in the allelic and homozygotic models, under the prior probability level of 0.1 (Table 6). There are only three case–control studies [36, 40, 43] in the Caucasian population in the pooling analysis. After removing one study with the HB-based control [36], only two studies with 460 cases and 921 controls were enrolled for the pooling analysis (Additional file 1: Table S3). Although we observed an increased risk of colorectal cancer under the homozygotic, heterozygotic and dominant models (Additional file 1: Table S3, *P *< 0.05, OR > 1), this does not exceed our minimum requirement for pooling analysis, which requires at least three case–control studies. We cannot obtain a relatively scientific conclusion regarding the potential links of *XPA* rs1800975 and colorectal cancer risk.

**Fig. 4:**
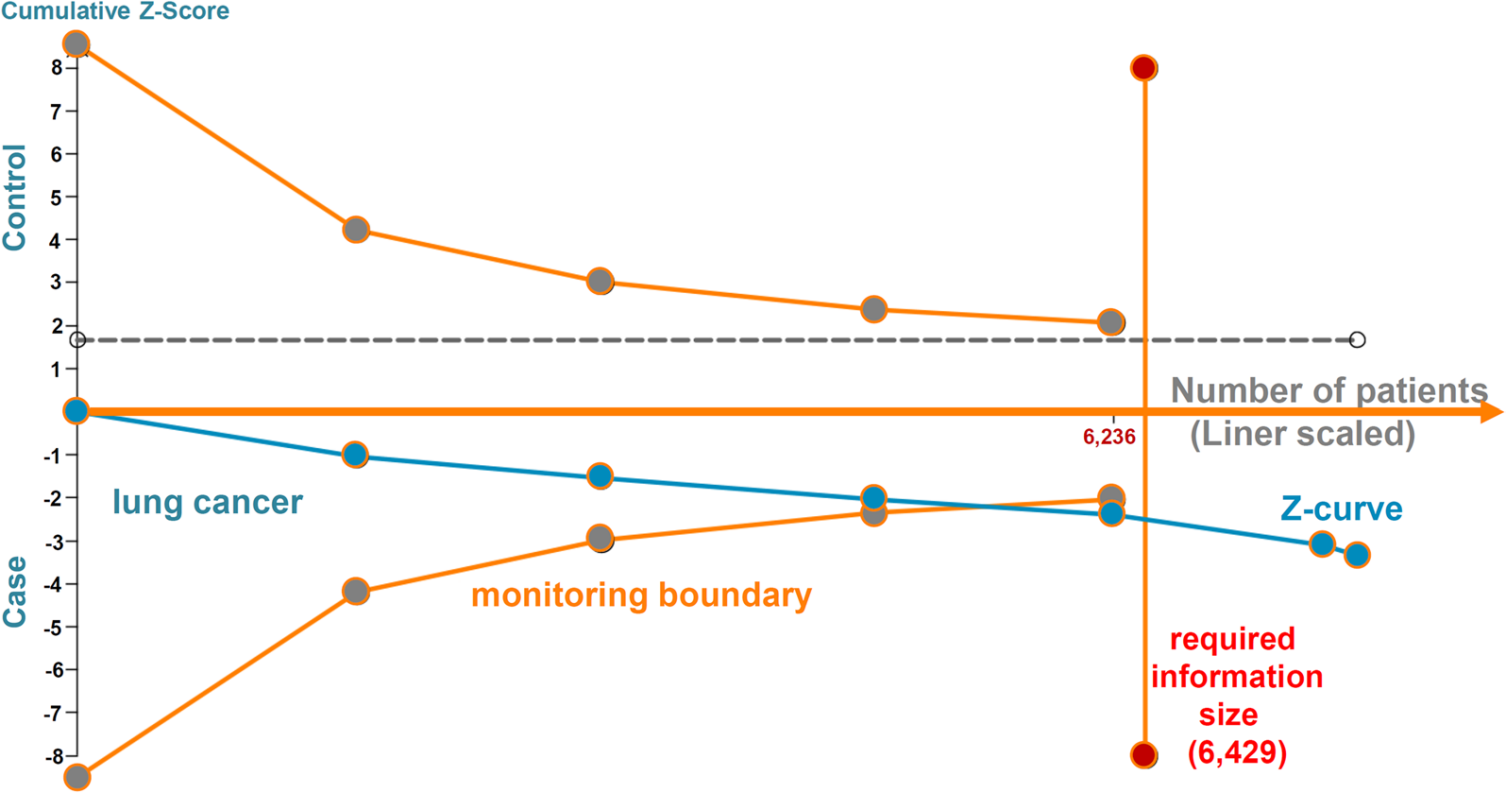
TSA plot for skin cancer under the dominant model

As shown in Table 6, under the 0.1 prior probability level, the FPRP values for skin cancer were all less than 0.20, confirming notable associations. Caucasian subjects and PB-based controls were enrolled in all case–control studies. We further performed the TSA test, and the TSA plot in Fig. 4 shows that the cumulative Z-curve of the dominant model can cross both the lines of the TSA monitoring boundary and the required information size, suggesting a credible conclusion regarding the association between *XPA* rs1800975 and skin susceptibility.

### Publication bias and sensitivity analysis results

**Fig. 5:**
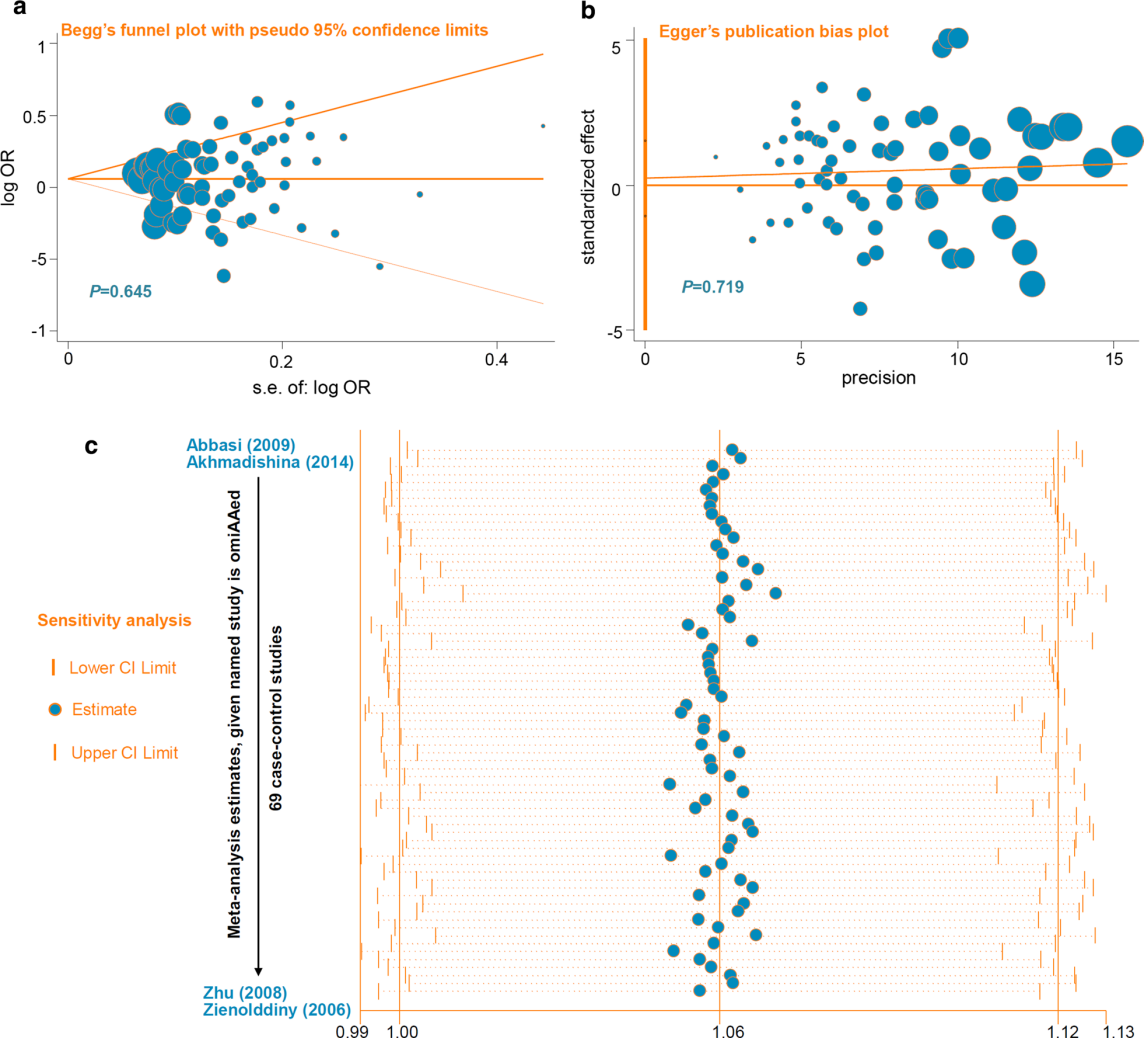
Publication bias and sensitivity analysis (allelic model). **a** Begg’s test data; **b** Egger’s test data; **c** sensitivity analysis data

For the evaluation of publication bias, the two-sided *P*-value of Begg’s and Egger’s test > 0.05 (Table 2) and the absence of obvious asymmetry of funnel plots under each genetic model (Fig. 5a, b show the plots of allelic model as instances) suggested no evidence of large publication bias during the pooling analysis mentioned above. In addition, we failed to detect the greatly changed values of ORs and 95% CIs through our leave-one-out sensitivity analysis (Fig. 5c for allelic model as an example).

### The eQTL and sQTL analysis results

**Fig. 6:**
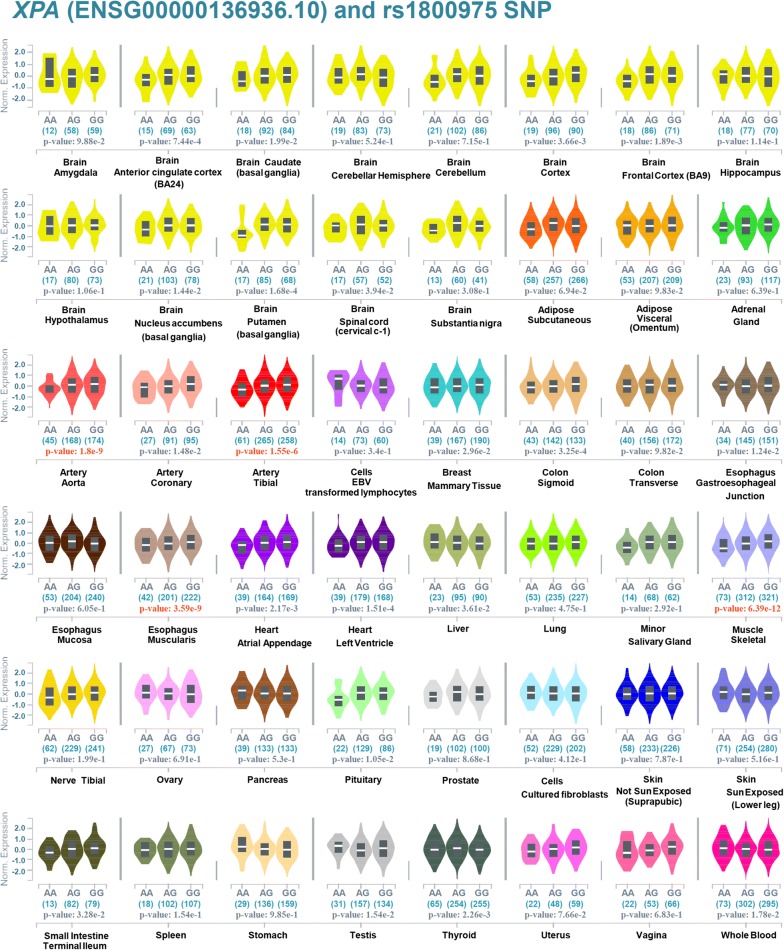
Violin plots of eQTL across multiple tissues of GTEx project

**Fig. 7:**
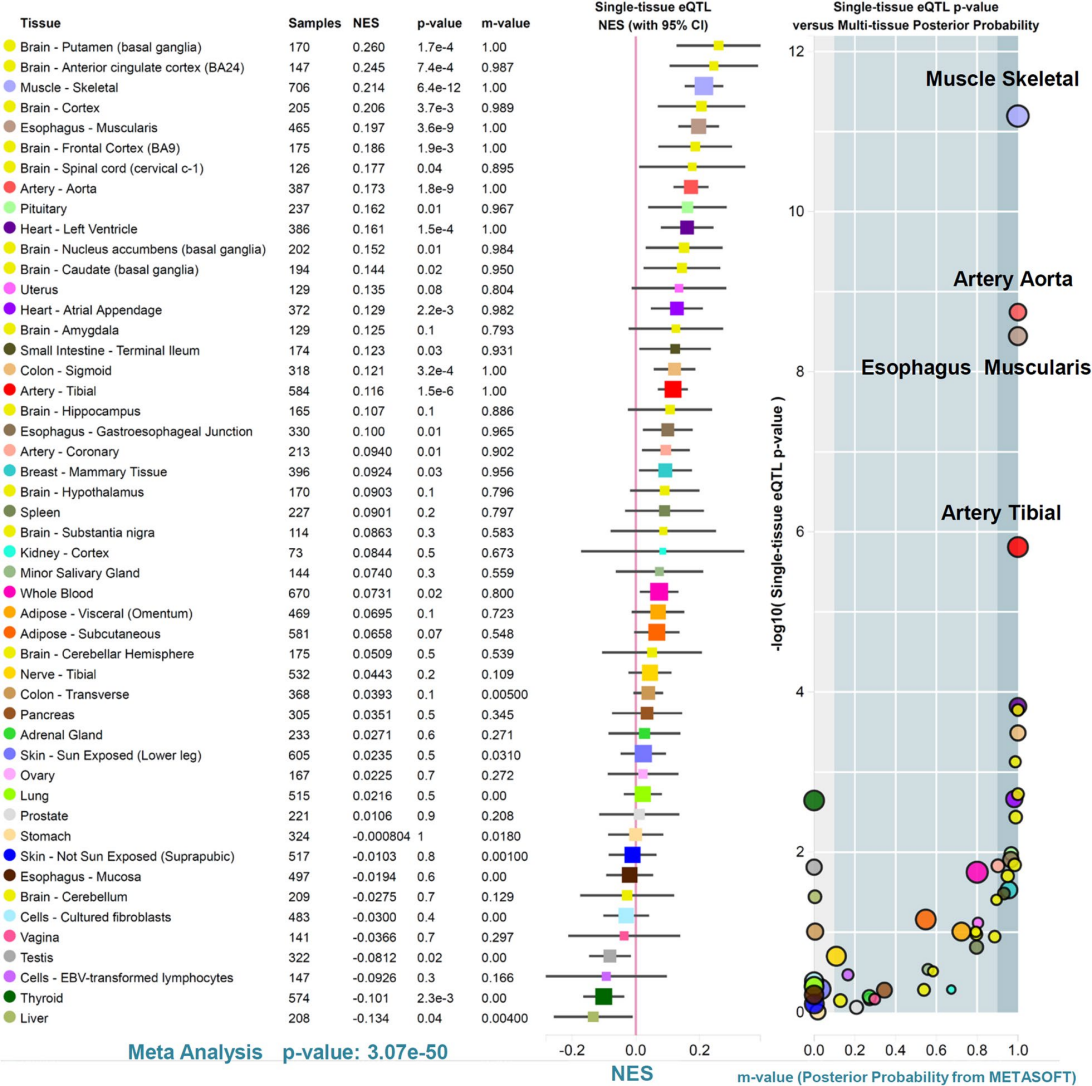
Multi-tissue eQTL plots of cross-tissue meta-analysis (GTEx)

**Fig. 8:**
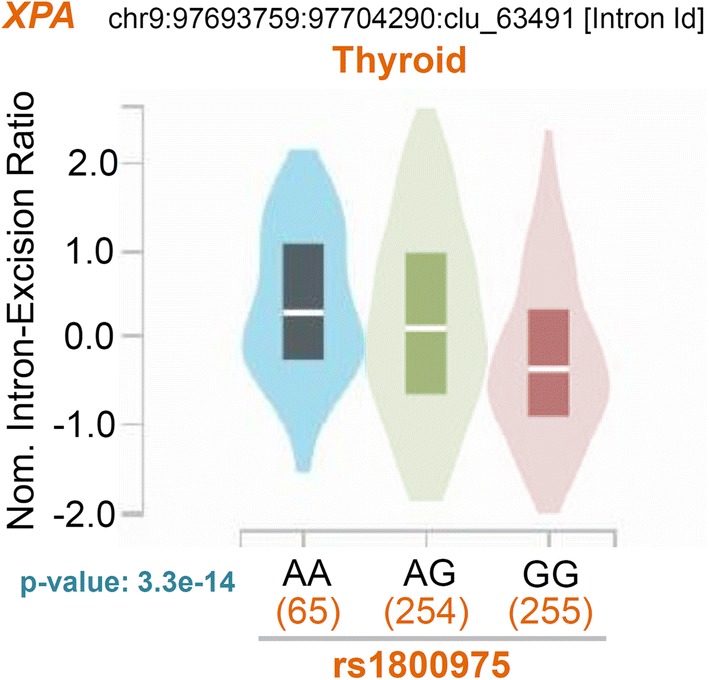
Violin plot of sQTL in the thyroid tissue of GTEx project

Finally, based on GTEx datasets, we analyzed the expression profile of the *XPA* gene in different tissues, and the correlation between the gene expression and rs1800975 SNP of *XPA*. As shown in Additional file 10: Fig. S9, the *XPA* gene is expressed in various tissues, such as the brain, colon, esophagus, lung or skin tissues, suggesting a low tissue specificity. Based on the “Significant Single-Tissue” eQTL data (Fig. 6), we observed the potential association between *XPA* gene expression and rs1800975 SNP, in the tissues of artery aorta (*P*-value = 1.8e−9), artery tibial (*P*-value = 1.55e−6), esophagus muscularis (*P*-value = 3.59e−9), muscle skeletal (*P*-value = 6.39e−12), but not the skin tissue of [“not sun exposed (suprapubic)”, *P*-value = 7.87e−1) or [“sun exposed (lower leg)”, *P*-value = 5.16e−1). The data of multi-tissue eQTL comparison also suggested that four tissues (artery aorta, artery tibial, esophagus muscularis, muscle skeletal) were predicted to have an eQTL effect (Fig. 7, all m-value = 1.00). Cross-tissue meta-analysis further showed a potential overall correlation between gene expression and rs1800975 SNP of *XPA* (Fig. 7, *P*-Value = 3.07e−50). In addition, our sQTL data further showed a potential association between rs1800975 SNP and the splicing changes of *XPA* gene in the thyroid tissue (Fig. 8).

## Discussion

Although we observed a group of publications regarding the influence of *XPA* rs1800975 on the risk of certain specific cancers, such as lung cancer [69, 70], head and neck cancer [71], breast cancer [72], and digestive system cancer [73, 74], the evaluation strategies, study number and statistical power differed. We were interested in comprehensively exploring the impact of *XPA* rs1800975 on overall cancer susceptibility by pooling all currently available evidence. To date, there are only two reported meta-analyses from 2012 [12, 13] describing the association between *XPA* rs1800975 and susceptibility to overall cancer diseases. In the current study, we searched six online electronic databases, including PubMed, EMBASE, Cochrane, CNKI, WANFANG and VIP, with the last retrieval on April 8, 2020, to include a total of 71 case–control studies. Based on six genetic models (allelic, carrier, homozygotic, heterozygotic, dominant and recessive), a series of overall meta-analyses and subgroup analyses using the factors of race, control source and genotyping method, were used to scientifically assess the association between *XPA* rs1800975 polymorphism and the risk of cancer. Additionally, Begg’s test and Egger’s test, sensitivity analysis, FPRP analysis and TSA test were conducted.

In 2012, Ding et al. included a total of thirty-six case–control or case-cohort studies from twenty-eight publications to conduct a meta-analysis for the genetic effect of *XPA* rs1800975 on the susceptibility to overall cancer [13]. They did not detect a positive conclusion in the overall meta-analysis but a significant difference between controls and cases in the “lung cancer” subgroup analysis under the homozygotic and recessive models, the “Asian” subgroup in the dominant models, and the “skin cancer” subgroup in the homozygotic, heterozygotic, dominant and recessive models. In our updated meta-analysis, we excluded three publications in which the genotypic distribution of the control group was not in line with the HWE principle [75–77] and one publication related to oral premalignant lesions [78]. We also replaced one publication [79] with another one [67]. In addition, we added a total of twenty-eight publications for our new pooled analysis. In 2012, Liu et al. included twenty-four publications to conduct another meta-analysis and reported an increased colorectal cancer risk under the homozygotic and dominant models but a decreased susceptibility to lung cancer under the homozygotic and dominant models [12]. In the present study, we removed two publications owing to HWE [75, 77] and added another thirty new publications for our updated integrative analysis.

Our new findings showed a positive conclusion in the overall meta-analysis only under the carrier and recessive models, and in the “Caucasian” subgroup analysis under each model. We failed to detect a significant difference between cases and controls in the Asian population. The sample size contributes to the inconsistency with the data of Ding et al. [13].

Additionally, we detected a decreased lung cancer risk in cases under the GG vs. AA, AG vs. AA, AG + GG vs. AA models but an increased risk of colorectal cancer under the allelic, homozygotic, heterozygotic, dominant models, indicating the possible effect of the AG genotype of *XPA* rs1800975 on the susceptibility to colorectal cancer. These findings are partly in line with the conclusion of the above prior meta-analyses [12, 13]. Nevertheless, our data from FPRP analysis and another pooling analysis with only the population-based controls in the Caucasian population did not strongly support the protective role of the G allele within the *XPA* rs1800975 polymorphism in the risk of lung or colorectal cancer. Our data from the pooling analysis, FPRP analysis and TSA demonstrated a significant difference between skin cancer cases and negative controls under six genetic models, suggesting the contribution of the G allele within *XPA* rs1800975 to an enhanced susceptibility to skin cancer. Our eQTL and sQTL analysis data of GTEx showed that the *XPA* rs1800975 might not be associated with the gene expression or splicing changes of *XPA* in the skin tissue, suggesting the existence of other molecular mechanisms.

There are several strengths within our pooling analysis. No case–control study with poor quality was enrolled. We also excluded studies in which the genotypic contribution in the control group was not in Hardy-Weinberg equilibrium. In addition, both the absence of larger publication bias and the stability of pooling data were observed in all comparisons.

There are also several disadvantages during our analyses, which need to be discussed. First, fewer than ten case–control studies were enrolled in some comparisons, such as the subgroup meta-analysis of “breast cancer”, “gastric cancer”, “colorectal cancer”, “endometrial cancer”, “head and neck cancer”, and “skin cancer”. Therefore, several comparisons, such as subgroup analyses of “oral cancer” or “skin SCC”, were not carried out. In addition, high heterogeneity was present, and the “random-effect with DerSimonian and Laird method” was set in the overall meta-analyses under the allelic, homozygotic, heterozygotic, dominant and recessive models. There exists a decreased level of between-study heterogeneity in some subgroups of “Caucasian” (data not shown), indicating that ethnicity may be involved in the heterogeneity source.

After investigating the expression difference of *XPA* gene between tumor and adjacent normal tissues in TCGA project (Additional file 11: Fig. S10), we observed a higher expression level of *XPA* in the tissues of CHOL (Cholangiocarcinoma, *P *< 0.001) and LIHC (Liver hepatocellular carcinoma, *P *< 0.001), but a lower level in the tissues of BLCA (Bladder Urothelial Carcinoma), BRCA (Breast invasive carcinoma), KICH (Kidney Chromophobe), KIRC (Kidney renal clear cell carcinoma), KIRP (Kidney renal papillary cell carcinoma), LUAD (Lung adenocarcinoma), LUSC (Lung squamous cell carcinoma), READ (Rectum adenocarcinoma), THCA (Thyroid carcinoma), and UCEC (Uterine Corpus Endometrial Carcinoma) (all *P *< 0.05), compared with the corresponding control tissues. Apart from that, we predicted that the tissues of artery aorta, artery tibial, esophagus muscularis, muscle skeletal have an eQTL effect, while the thyroid tissue has a sQTL effect. Thus, it is meaningful to explore the potential genetic influence of all *XPA* genetic variants or the combined variants of *XPA* and other relevant genes (such as xeroderma pigmentosum group D, *XPD*) in the pathogenesis of the above tumors, arterial or muscular system-related diseases. The larger sample sizes are warranted, and the factors of age, sex, smoking, drinking, or therapy should be adjusted.

## Conclusions

To summarize, our comprehensive integrative analysis data demonstrated statistical evidence on the association between the *XPA* rs1800975 A/G polymorphism and susceptibility to skin cancer, especially skin BCC, in the Caucasian population. The enrollment of more case–control studies following the HWE principle in diverse ethnicities will help researchers to further verify the potential genetic role of the *XPA* rs1800975 polymorphism in the risk of lung or colorectal cancer.

## Supplementary information

**Additional file 1: Table S1.** Search terms of six online databases. **Table S2.** The association between *XPA* rs1800975 and the risk of lung cancers in the Caucasian population. **Table S3.** The association between *XPA* rs1800975 and the risk of lung and colorectal cancers in the Caucasian population (only PB-based controls).

**Additional file 2: Fig. S1.** Forest plot data of subgroup analysis by race (carrier model).

**Additional file 3: Fig. S2.** Forest plot data of subgroup analysis by race (dominant model).

**Additional file 4: Fig. S3.** Forest plot data of subgroup analysis by control source (allelic model).

**Additional file 5: Fig. S4.** Forest plot data of subgroup analysis by control source (carrier model).

**Additional file 6: Fig. S5.** Forest plot data of subgroup analysis by control source (dominant model).

**Additional file 7: Fig. S6.** Forest plot data of subgroup analysis by cancer type (homozygotic model).

**Additional file 8: Fig. S7.** Forest plot data of subgroup analysis by cancer type (heterozygotic model).

**Additional file 9: Fig. S8.** Forest plot data of subgroup analysis by cancer type (dominant model).

**Additional file 10: Fig. S9.** Violin plot of *XPA* expression profile across multiple tissues of GTEx project.

**Additional file 11: Fig. S10.** Box plot of the expression difference of *XPA* gene between tumor and adjacent normal tissues across all TCGA tumors. ** *P*<0.01; *** *P*<0.001.

## Data Availability

All data generated or analyzed during the present study are included in this published article.

## Abbreviations

XPA: Xeroderma pigmentosum group A
OR: Odd ratio
FPRP: False-positive report probability
TSA: Trial sequential analysis
eQTL: Expression quantitative trait loci
sQTL: Splicing quantitative trait loci
BCC: Basal cell carcinoma
NER: Nucleotide excision repair
SNP: Single nucleotide polymorphism
PRISMA: Preferred reporting items for systematic reviews and meta-analyses
HWE: Hardy–Weinberg equilibrium
EMBASE: Excerpta Medica Database
CNKI: China National Knowledge Infrastructure
NOS: Newcastle–Ottawa quality assessment Scale
CI: Confidence interval
GTEx: Genotype-Tissue Expression
TPM: Transcripts Per Million
TIMER: Tumor Immune Estimation Resource
TCGA: The Cancer Genome Atlas
NES: Normalized Effect Size
CHOL: Cholangiocarcinoma
LIHC: Liver hepatocellular carcinoma
BLCA: Bladder Urothelial Carcinoma
BRCA: Breast invasive carcinoma
KICH: Kidney Chromophobe
KIRC: Kidney renal clear cell carcinoma
KIRP: Kidney renal papillary cell carcinoma
LUAD: Lung adenocarcinoma
LUSC: Lung squamous cell carcinoma
READ: Rectum adenocarcinoma
THCA: Thyroid carcinoma
UCEC: Uterine Corpus Endometrial Carcinoma
XPD: Xeroderma pigmentosum group D
BC: Bladder cancer
RCC: Renal cell carcinoma
SCC: Squamous cell carcinoma
LSCC: Lung squamous cell carcinoma
GCA: Gastric cardiac adenocarcinoma
LA: Lung adenocarcinoma
NSCLC: Non-small cell lung cancer
ESCC: Esophageal squamous cell carcinoma
OSCC: Oral squamous cell carcinoma
ALL: Acute lymphoblastic leukemia
HCC: Hepatocellular carcinoma
PB: Population-based control
HB: Hospital-based control
PCR: Polymerase chain reaction
PCR-RFLP: PCR-restriction fragment length polymorphism
PCR-LDR: PCR-ligase detection reaction
MALDI-TOF-MS: Matrix-assisted laser desorption/Ionization time of flight mass spectrometry
