# Supplementary material for: Association of human XPA rs1800975 polymorphism and cancer susceptibility: an integrative analysis of 71 case–control studies
Source: Cancer Cell Int. 2020 May 13;20:164. doi: 10.1186/s12935-020-01244-5 (PMC7218628; doi:10.1186/s12935-020-01244-5)
Supplement: Supplementary file 1 — Additional file 1: Table S1. Search terms of six online databases. Table S2. The association between XPA rs1800975 and the risk of lung cancers in the Caucasian population. Table S3. The association between XPA rs1800975 and the risk of lung and colorectal cancers in the Caucasian population (only PB-based controls). [file 12935_2020_1244_MOESM1_ESM.docx]

### Table S1 Search terms of six online databases.

| **step** | **term** | **Number** |
| --- | --- | --- |
| **（1）PubMed** | | |
| step1 | (((((((((((((((((Neoplasms) OR Neoplasia) OR Neoplasias) OR Neoplasm) OR Tumors) OR Tumor) OR Cancer) OR Cancers) OR Malignancy) OR Malignancies) OR Malignant Neoplasms) OR Malignant Neoplasm) OR Neoplasm, Malignant) OR Neoplasms, Malignant) OR Benign Neoplasms) OR Neoplasms, Benign) OR Benign Neoplasm) OR Neoplasm, Benign | 4,575,413 |
| step 2 | (((((((Xeroderma Pigmentosum Group A) OR (XPA, DNA Damage Recognition And Repair Factor)) OR Xeroderma Pigmentosum Group A-Complementing Protein) OR Xeroderma Pigmentosum, Complementation Group A) OR DNA Repair Protein Complementing XP-A Cells) OR XPAC) OR XP1) OR XPA | 1,264 |
| step 3 | (((((((((Polymorphism, Single Nucleotide) OR Nucleotide Polymorphism, Single) OR Nucleotide Polymorphisms, Single) OR Polymorphisms, Single Nucleotide) OR Single Nucleotide Polymorphisms) OR SNPs) OR SNP) OR Single Nucleotide Polymorphism) OR A23G) OR rs1800975 | 158,167 |
| step 4 | ((((((((((((((((((Neoplasms) OR Neoplasia) OR Neoplasias) OR Neoplasm) OR Tumors) OR Tumor) OR Cancer) OR Cancers) OR Malignancy) OR Malignancies) OR Malignant Neoplasms) OR Malignant Neoplasm) OR Neoplasm, Malignant) OR Neoplasms, Malignant) OR Benign Neoplasms) OR Neoplasms, Benign) OR Benign Neoplasm) OR Neoplasm, Benign) AND ((((((((Xeroderma Pigmentosum Group A) OR (XPA, DNA Damage Recognition And Repair Factor)) OR Xeroderma Pigmentosum Group A-Complementing Protein) OR Xeroderma Pigmentosum, Complementation Group A) OR DNA Repair Protein Complementing XP-A Cells) OR XPAC) OR XP1) OR XPA) AND ((((((((((Polymorphism, Single Nucleotide) OR Nucleotide Polymorphism, Single) OR Nucleotide Polymorphisms, Single) OR Polymorphisms, Single Nucleotide) OR Single Nucleotide Polymorphisms) OR SNPs) OR SNP) OR Single Nucleotide Polymorphism) OR A23G) OR rs1800975) | 124 |
| **（2）EMBASE** | | |
| step1 | 'neoplasm'/exp OR 'neoplasm' OR 'acral tumor'/exp OR 'acral tumor' OR 'acral tumour'/exp OR 'acral tumour' OR 'neoplasia'/exp OR 'neoplasia' OR 'neoplasms'/exp OR 'neoplasms' OR 'neoplasms by histologic type'/exp OR 'neoplasms by histologic type' OR 'neoplasms, cystic, mucinous, and serous'/exp OR 'neoplasms, cystic, mucinous, and serous' OR 'neoplasms, embryonal and mixed'/exp OR 'neoplasms, embryonal and mixed' OR 'neoplasms, germ cell and embryonal'/exp OR 'neoplasms, germ cell and embryonal' OR 'neoplasms, glandular and epithelial'/exp OR 'neoplasms, glandular and epithelial' OR 'neoplasms, hormone-dependent'/exp OR 'neoplasms, hormone-dependent' OR 'neoplasms, post-traumatic'/exp OR 'neoplasms, post-traumatic' OR 'neoplastic disease'/exp OR 'neoplastic disease' OR 'tumor'/exp OR 'tumor' OR 'tumour'/exp OR 'tumour' | 5,716,720 |
| step 2 | 'xeroderma pigmentosum group a'/exp OR 'xeroderma pigmentosum group a' OR 'xpa' OR 'xpac' OR 'xeroderma pigmentosum a' OR 'xeroderma pigmentosum complementation group a' | 1,880 |
| step 3 | 'single nucleotide polymorphism'/exp OR 'single nucleotide polymorphism' OR 'nucleotide polymorphism, single' OR 'polymorphism, single nucleotide'/exp OR 'polymorphism, single nucleotide' OR 'single nucleotide variant'/exp OR 'single nucleotide variant' OR 'single nucleotide variation'/exp OR 'single nucleotide variation' OR 'snp'/exp OR 'snp' OR 'a23g' OR 'rs1800975' | 211,499 |
| step 4 | ('neoplasm'/exp OR 'neoplasm' OR 'acral tumor'/exp OR 'acral tumor' OR 'acral tumour'/exp OR 'acral tumour' OR 'neoplasia'/exp OR 'neoplasia' OR 'neoplasms'/exp OR 'neoplasms' OR 'neoplasms by histologic type'/exp OR 'neoplasms by histologic type' OR 'neoplasms, cystic, mucinous, and serous'/exp OR 'neoplasms, cystic, mucinous, and serous' OR 'neoplasms, embryonal and mixed'/exp OR 'neoplasms, embryonal and mixed' OR 'neoplasms, germ cell and embryonal'/exp OR 'neoplasms, germ cell and embryonal' OR 'neoplasms, glandular and epithelial'/exp OR 'neoplasms, glandular and epithelial' OR 'neoplasms, hormone-dependent'/exp OR 'neoplasms, hormone-dependent' OR 'neoplasms, post-traumatic'/exp OR 'neoplasms, post-traumatic' OR 'neoplastic disease'/exp OR 'neoplastic disease' OR 'tumor'/exp OR 'tumor' OR 'tumour'/exp OR 'tumour') AND ('xeroderma pigmentosum group a'/exp OR 'xeroderma pigmentosum group a' OR 'xpa' OR 'xpac' OR 'xeroderma pigmentosum a' OR 'xeroderma pigmentosum complementation group a') AND ('single nucleotide polymorphism'/exp OR 'single nucleotide polymorphism' OR 'nucleotide polymorphism, single' OR 'polymorphism, single nucleotide'/exp OR 'polymorphism, single nucleotide' OR 'single nucleotide variant'/exp OR 'single nucleotide variant' OR 'single nucleotide variation'/exp OR 'single nucleotide variation' OR 'snp'/exp OR 'snp' OR 'a23g' OR 'rs1800975' ) | 147 |
| **（3）Cochrane** | | |
| step1 | (Xeroderma Pigmentosum Group A):ti,ab,kw OR (XPA, DNA Damage Recognition And Repair Factor):ti,ab,kw OR (Xeroderma Pigmentosum Group A-Complementing Protein):ti,ab,kw OR (Xeroderma Pigmentosum, Complementation Group A):ti,ab,kw OR (XPA):ti,ab,kw | 41 |
| step 2 | (Neoplasm):ti,ab,kw OR (Tumor):ti,ab,kw OR (Cancer):ti,ab,kw OR (Malignancy):ti,ab,kw OR (Neoplasia):ti,ab,kw | 197,578 |
| step 3 | ((Xeroderma Pigmentosum Group A):ti,ab,kw OR (XPA, DNA Damage Recognition And Repair Factor):ti,ab,kw OR (Xeroderma Pigmentosum Group A-Complementing Protein):ti,ab,kw OR (Xeroderma Pigmentosum, Complementation Group A):ti,ab,kw OR (XPA):ti,ab,kw) AND ((Neoplasm):ti,ab,kw OR (Tumor):ti,ab,kw OR (Cancer):ti,ab,kw OR (Malignancy):ti,ab,kw OR (Neoplasia):ti,ab,kw) | 33 |
| **（4）CNKI** | | |
| step1 | SU= 'XPA' or SU='polymorphism ' | 13 |
| **（5）WANFANG** | | |
| step1 | XPA * polymorphism | 74 |
| **（6）VIP** | | |
| step1 | XPA / polymorphism | 9 |

*EMBASE* Excerpta Medica Database, *CNKI* China National Knowledge Infrastructure

### Table S2 The association between *XPA* rs1800975 and the risk of lung cancers in the Caucasian population.

| **Model** | **Study number (case/control)** | **OR (95% CI)** | **z** | ***P*** |
| --- | --- | --- | --- | --- |
| **Homozygotic model (GG vs. AA)** | 7(2,172/3,757) | 0.97(0.78-1.20) | 0.31 | 0.757 |
| **Heterozygotic model (AG vs. AA)** | 7(2,172/3,757) | 0.95(0.80-1.14) | 0.55 | 0.585 |
| **Dominant model (AG+GG vs. AA)** | 7(2,172/3,757) | 0.97(0.82-1.15) | 0.35 | 0.728 |

*OR* odds ratio, *CI* confidence interval

### Table S3 The association between *XPA* rs1800975 and the risk of lung and colorectal cancers in the Caucasian population (only PB-based controls).

| **Cancer type** | **Model** | **Study number (case/control)** | **OR (95% CI)** | **z** | ***P*** |
| --- | --- | --- | --- | --- | --- |
| **Lung cancer** | Homozygotic model (GG vs. AA) | 5(1,758/2,843) | 1.01(0.77-1.34) | 0.10 | 0.920 |
|  | Heterozygotic model (AG vs. AA) | 5(1,758/2,843) | 1.02(0.83-1.24) | 0.17 | 0.869 |
|  | Dominant model (AG+GG vs. AA) | 5(1,758/2,843) | 1.03(0.85-1.25) | 0.30 | 0.765 |
| **Colorectal cancer** | Allelic model (G vs. A) | 2(460/921) | 1.16(0.98-1.38) | 1.77 | 0.077 |
|  | Homozygotic model (GG vs. AA) | 2(460/921) | 1.62(1.07-2.46) | 2.26 | 0.024 |
|  | Heterozygotic model (AG vs. AA) | 2(460/921) | 1.57(1.04-2.38) | 2.13 | 0.033 |
|  | Dominant model (AG+GG vs. AA) | 2(460/921) | 1.60(1.07-2.38) | 2.29 | 0.022 |

*OR* odds ratio, *CI* confidence interval, *PB* population-based control
