# Supplementary figures and images for: Association of human XPA rs1800975 polymorphism and cancer susceptibility: an integrative analysis of 71 case–control studies
Source: Cancer Cell Int. 2020 May 13;20:164. doi: 10.1186/s12935-020-01244-5 (PMC7218628; doi:10.1186/s12935-020-01244-5)

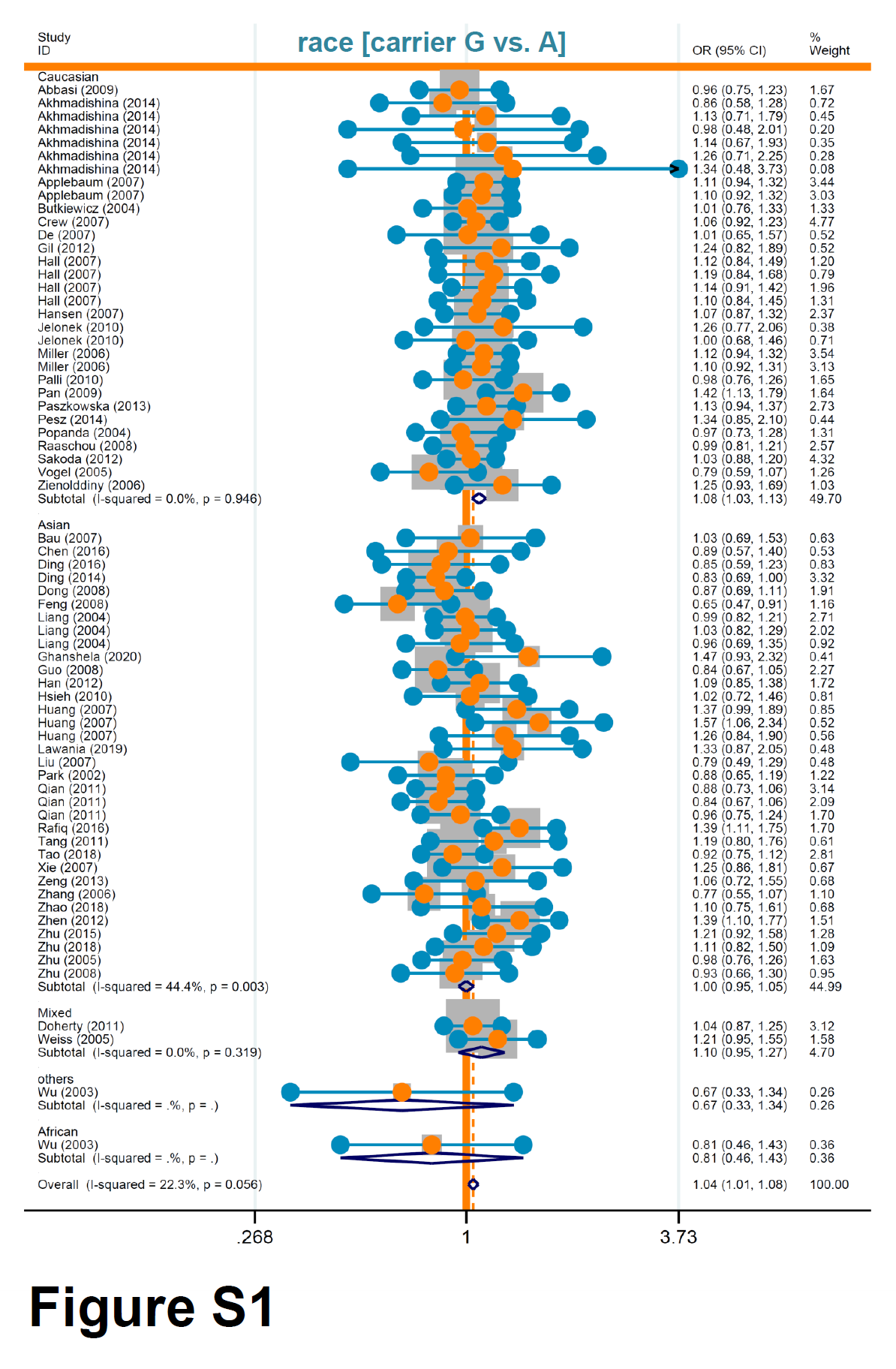

Supplement: Supplementary file 2 — Additional file 2: Fig. S1. Forest plot data of subgroup analysis by race (carrier model). [file 12935_2020_1244_MOESM2_ESM.tif]

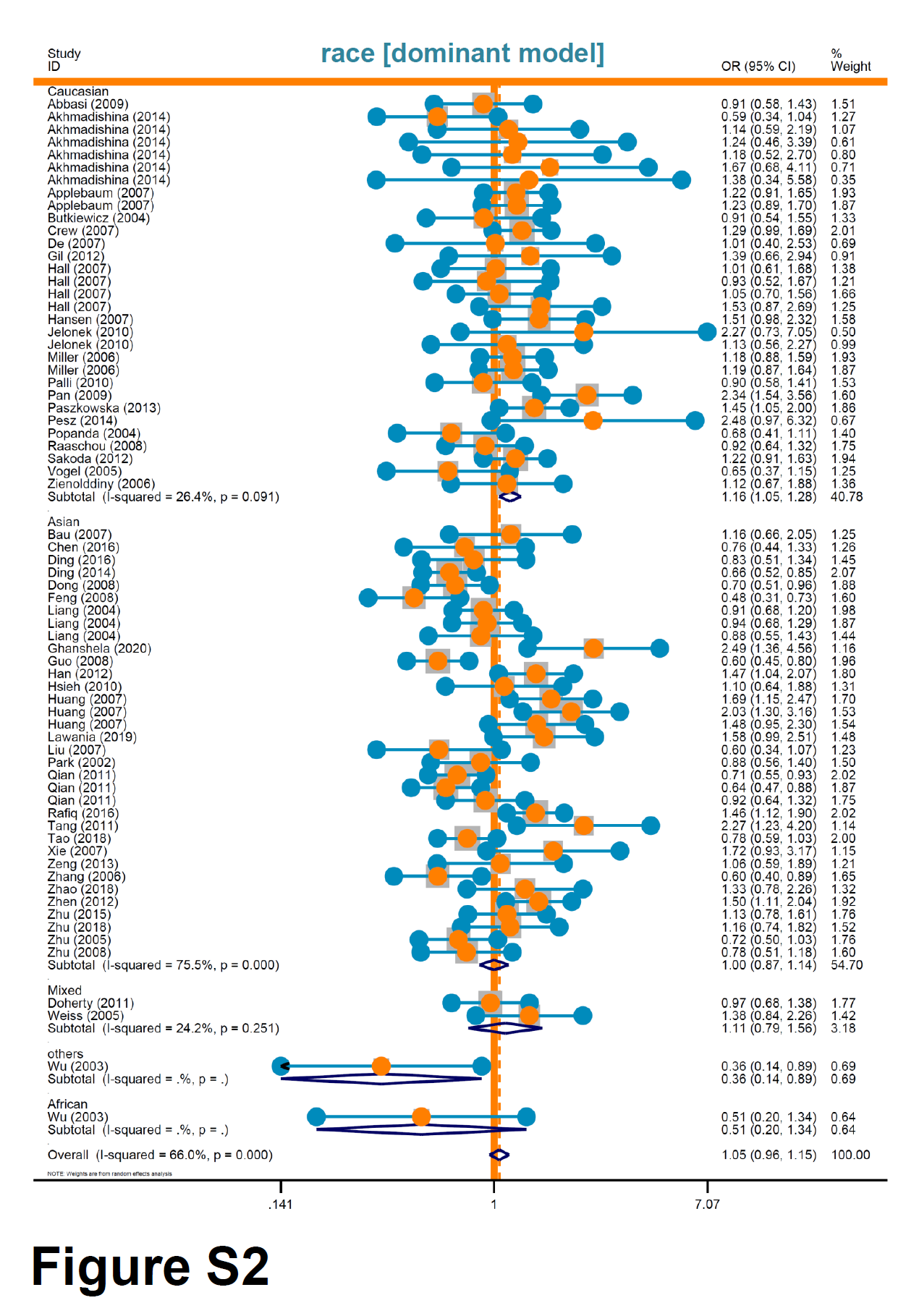

Supplement: Supplementary file 3 — Additional file 3: Fig. S2. Forest plot data of subgroup analysis by race (dominant model). [file 12935_2020_1244_MOESM3_ESM.tif]

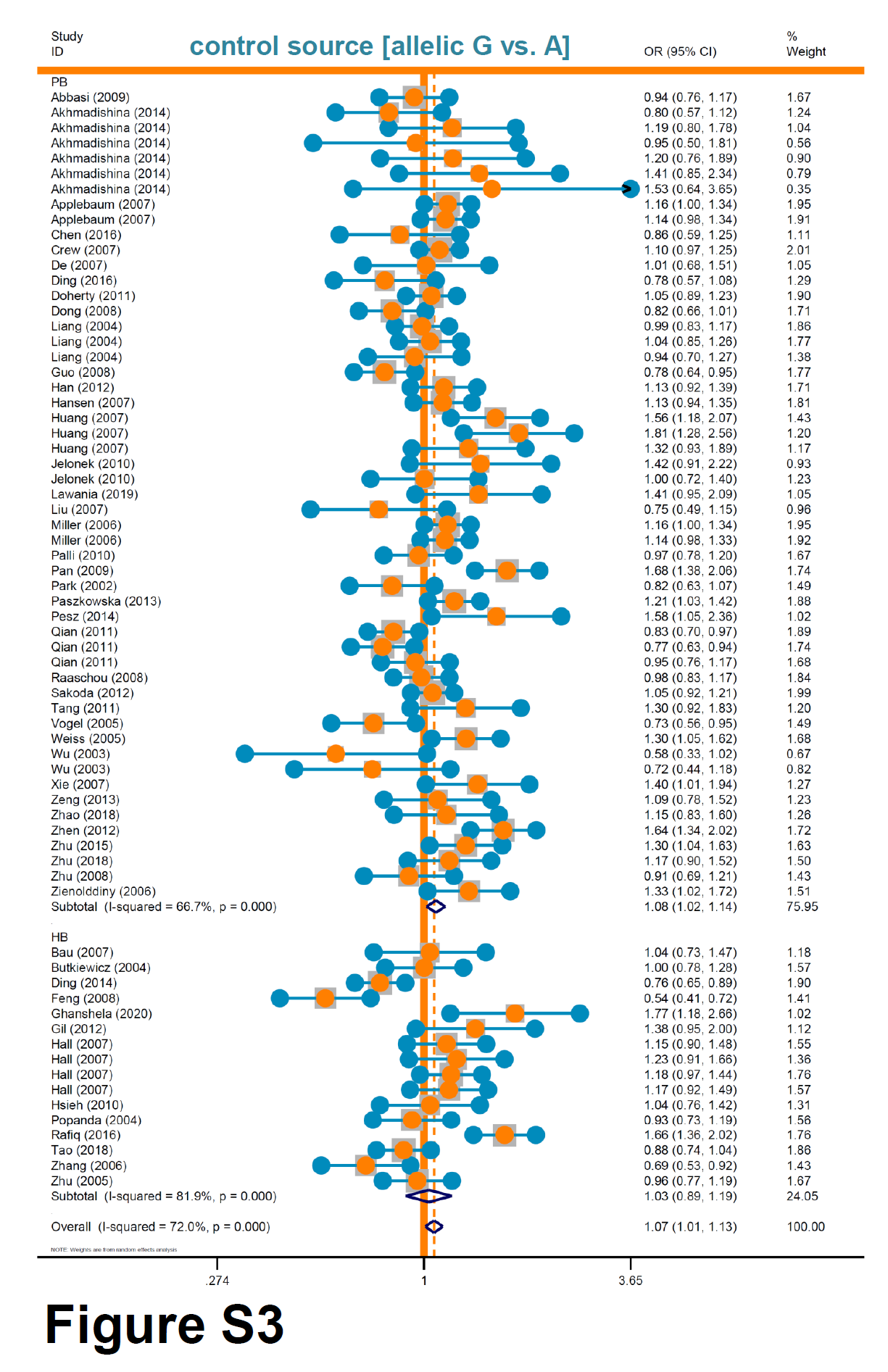

Supplement: Supplementary file 4 — Additional file 4: Fig. S3. Forest plot data of subgroup analysis by control source (allelic model). [file 12935_2020_1244_MOESM4_ESM.tif]

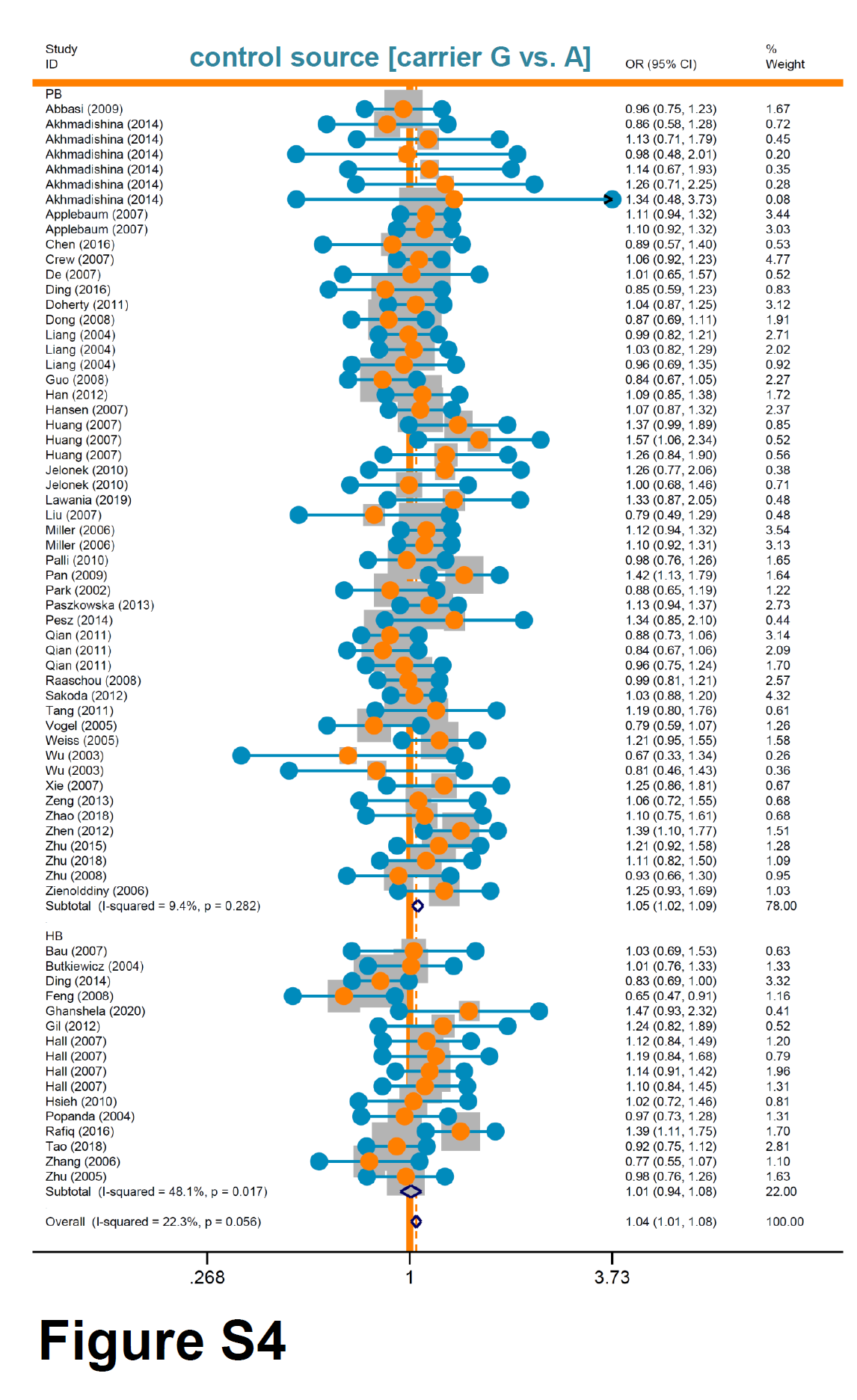

Supplement: Supplementary file 5 — Additional file 5: Fig. S4. Forest plot data of subgroup analysis by control source (carrier model). [file 12935_2020_1244_MOESM5_ESM.tif]

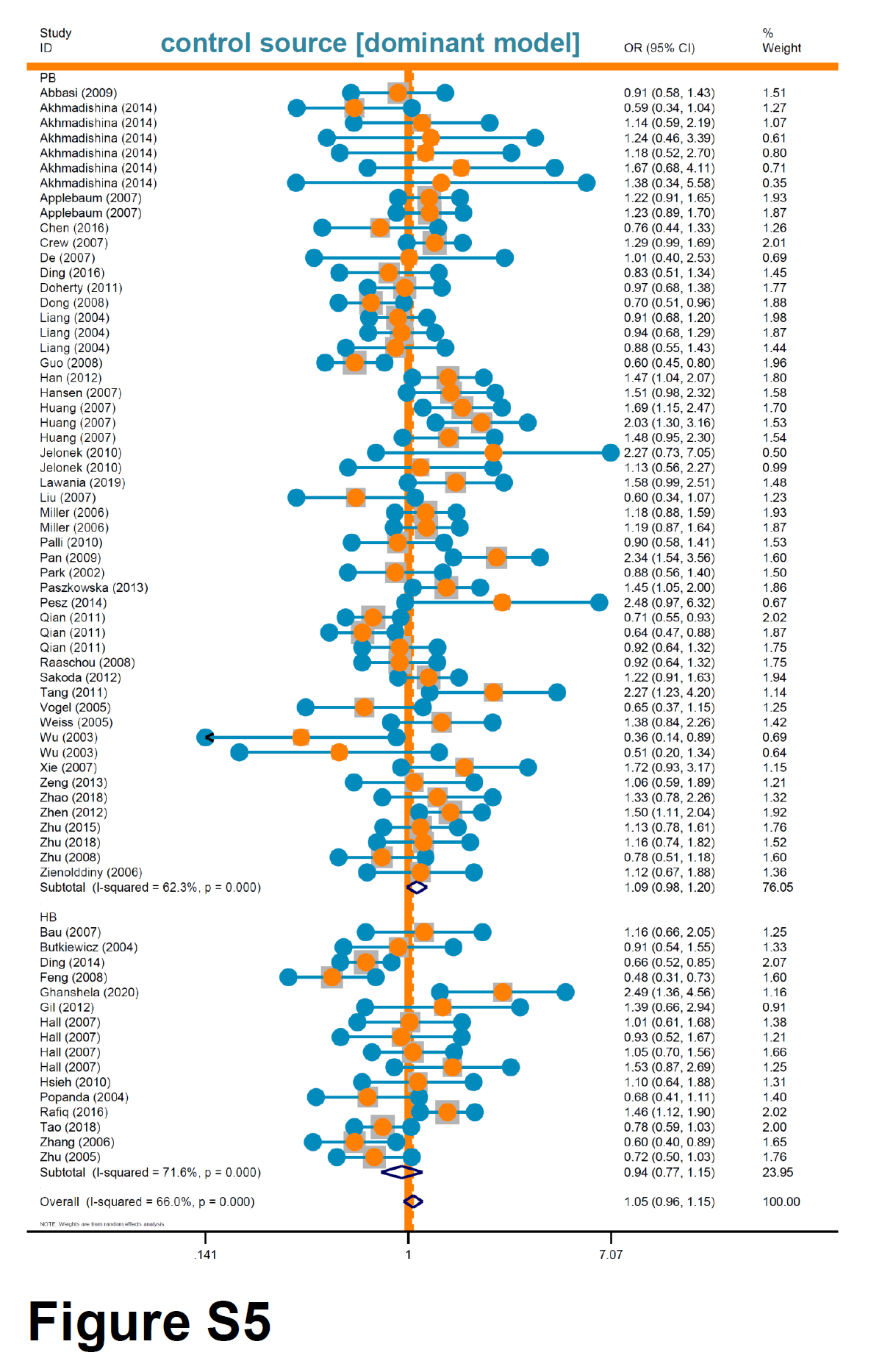

Supplement: Supplementary file 6 — Additional file 6: Fig. S5. Forest plot data of subgroup analysis by control source (dominant model). [file 12935_2020_1244_MOESM6_ESM.tif]

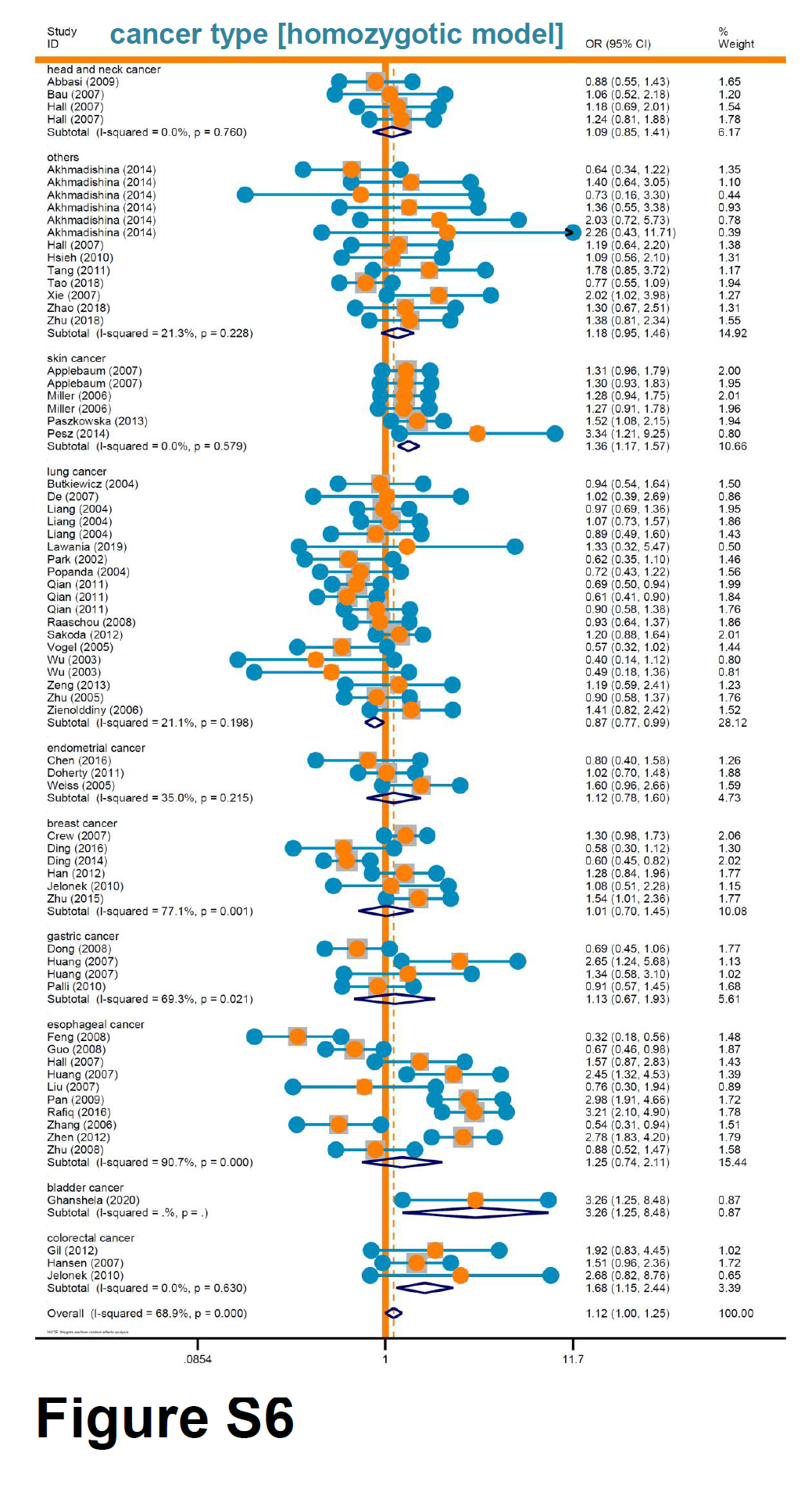

Supplement: Supplementary file 7 — Additional file 7: Fig. S6. Forest plot data of subgroup analysis by cancer type (homozygotic model). [file 12935_2020_1244_MOESM7_ESM.tif]

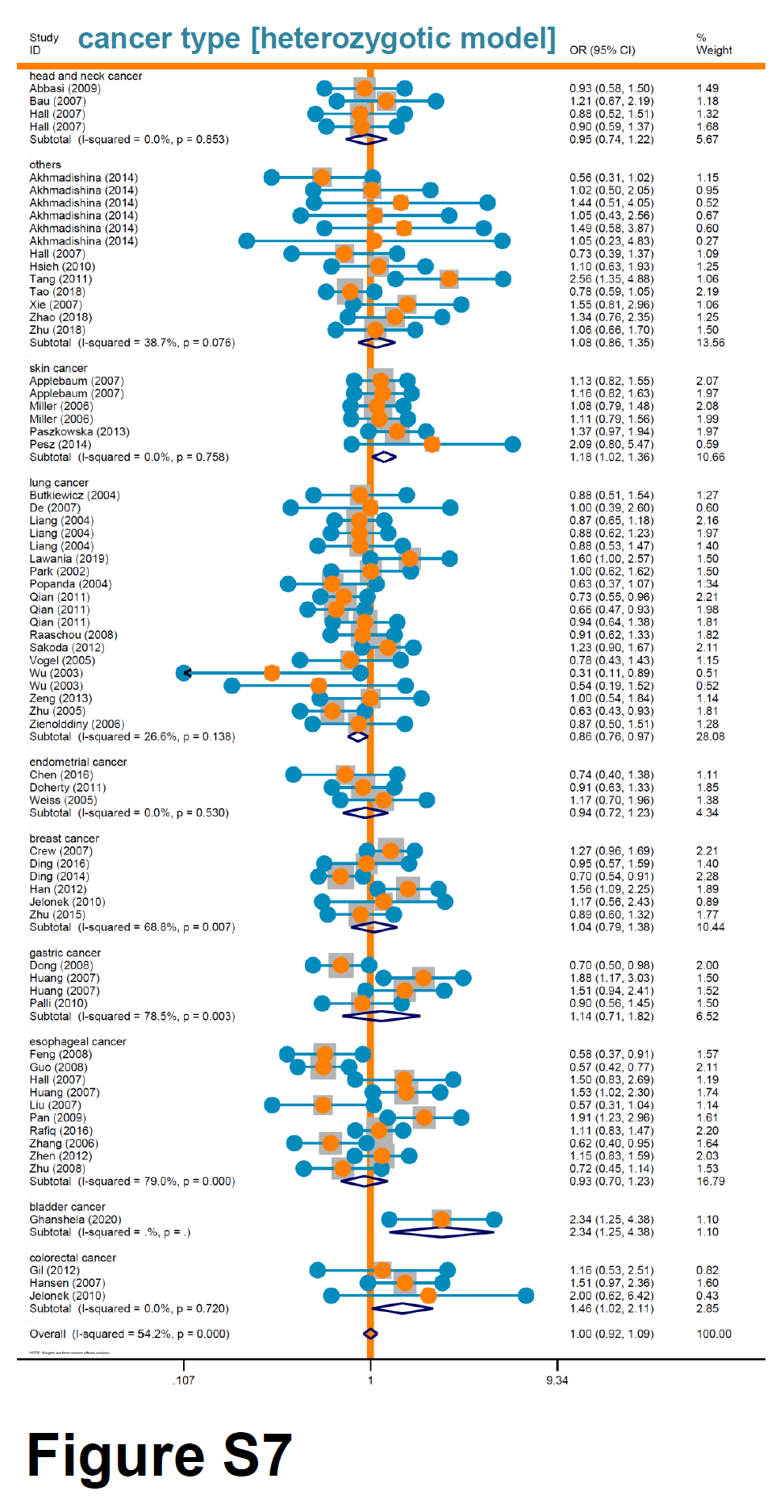

Supplement: Supplementary file 8 — Additional file 8: Fig. S7. Forest plot data of subgroup analysis by cancer type (heterozygotic model). [file 12935_2020_1244_MOESM8_ESM.tif]

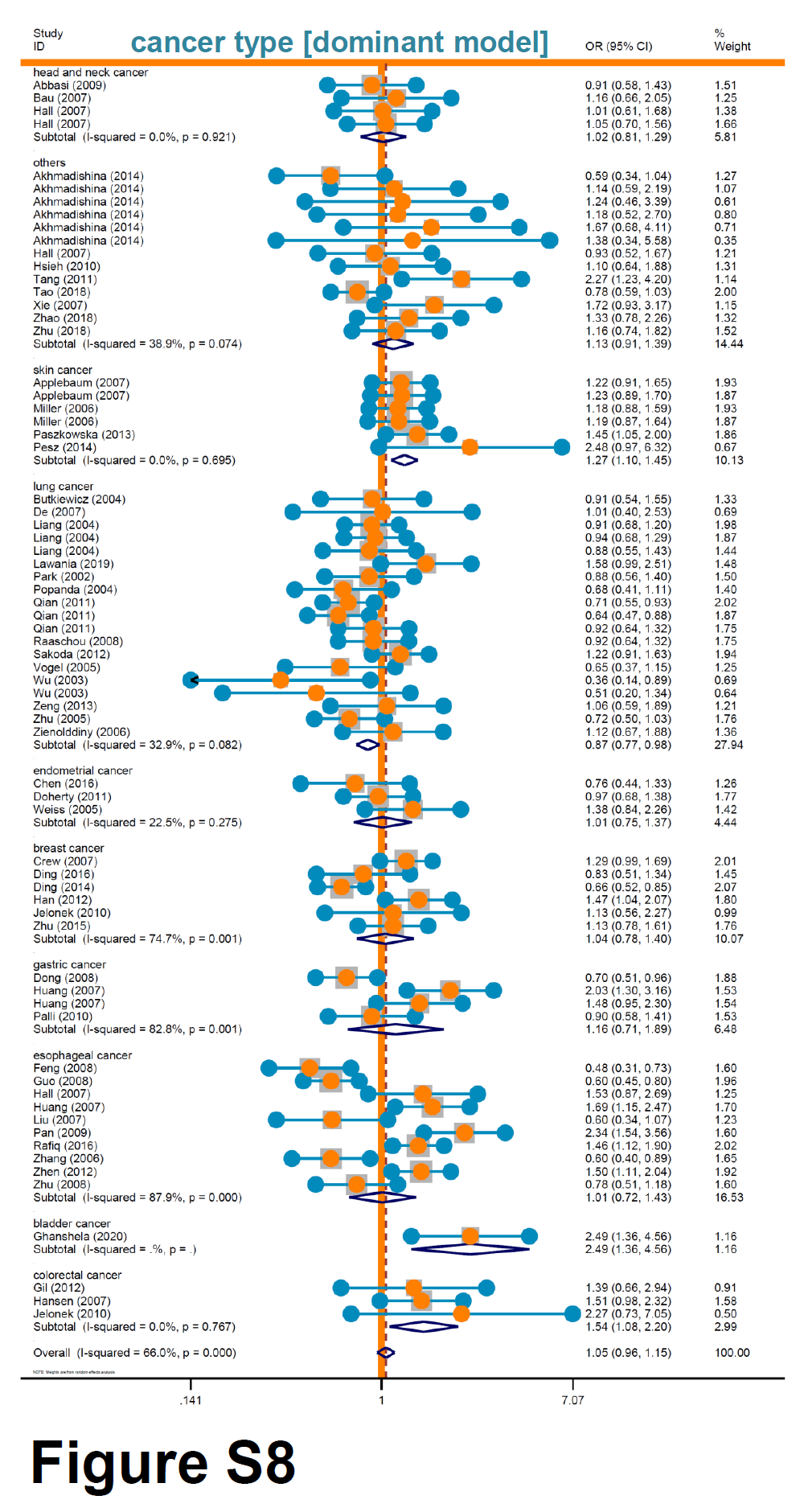

Supplement: Supplementary file 9 — Additional file 9: Fig. S8. Forest plot data of subgroup analysis by cancer type (dominant model). [file 12935_2020_1244_MOESM9_ESM.tif]

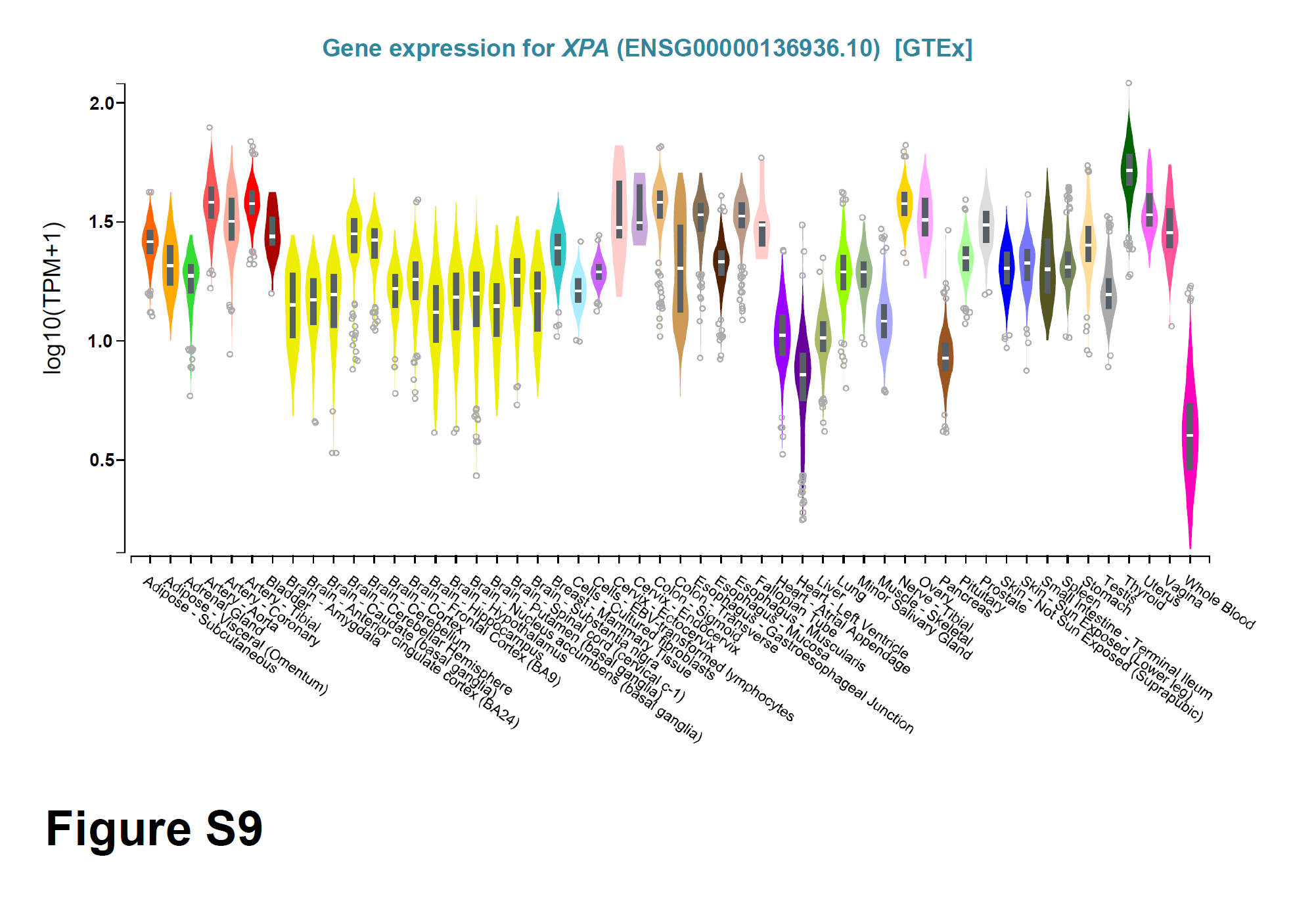

Supplement: Supplementary file 10 — Additional file 10: Fig. S9. Violin plot of XPA expression profile across multiple tissues of GTEx project. [file 12935_2020_1244_MOESM10_ESM.tif]

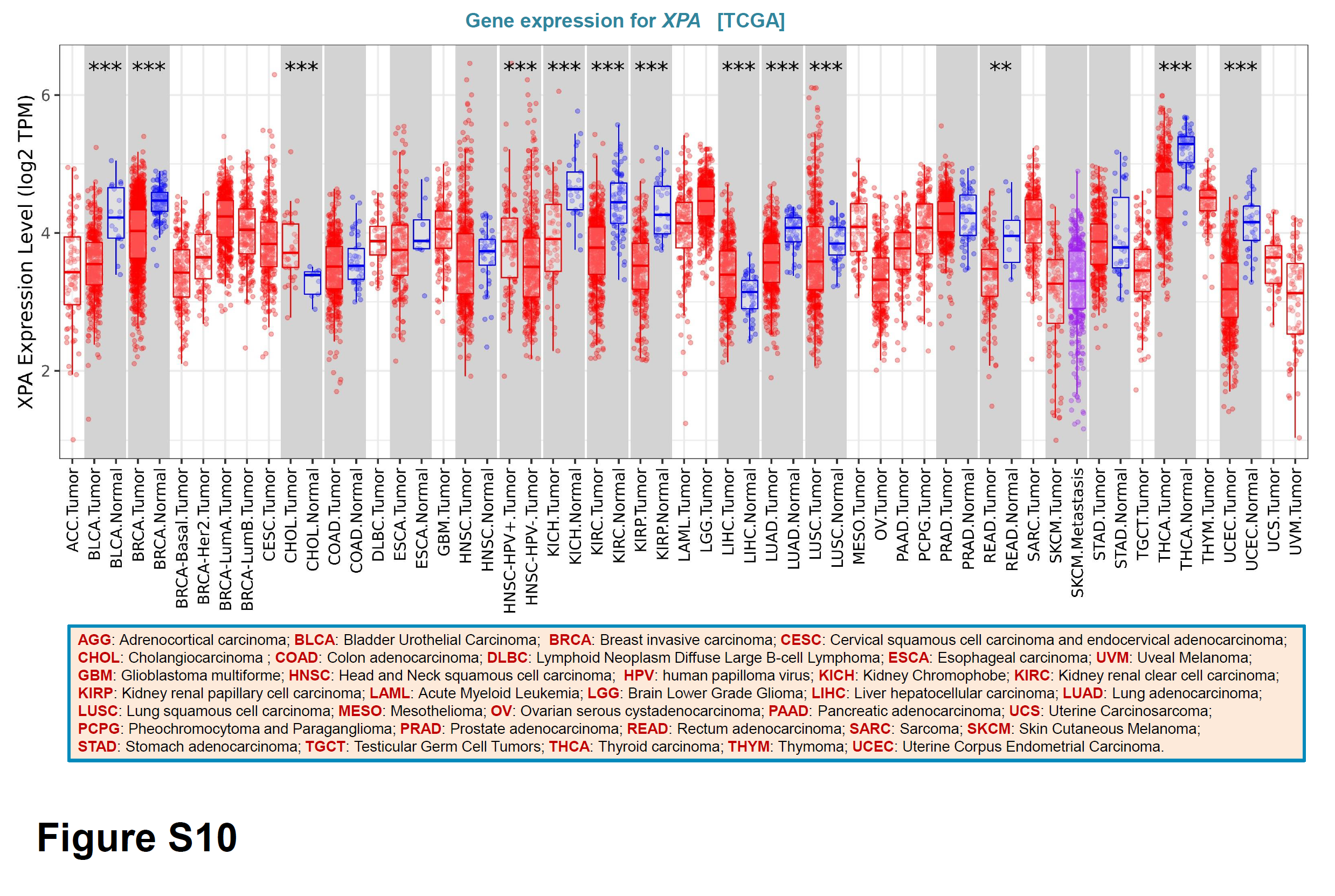

Supplement: Supplementary file 11 — Additional file 11: Fig. S10. Box plot of the expression difference of XPA gene between tumor and adjacent normal tissues across all TCGA tumors. ** P<0.01; *** P<0.001. [file 12935_2020_1244_MOESM11_ESM.tif]
